# Development of an integrated microperfusion-EEG electrode for unbiased multimodal sampling of brain interstitial fluid and concurrent neural activity

**DOI:** 10.1088/1741-2552/acad29

**Published:** 2023-01-18

**Authors:** Luke A Stangler, Evan N Nicolai, Filip Mivalt, Su-Youne Chang, Inyong Kim, Abbas Z Kouzani, Kevin Bennet, Michael Berk, Susheil Uthamaraj, Terry C Burns, Gregory A Worrell, Charles L Howe

**Affiliations:** 1 School of Engineering, Deakin University, Geelong, Victoria 3216, Australia; 2 Division of Engineering, Mayo Clinic, Rochester, MN 55905, United States of America; 3 Mayo Clinic Graduate School of Biomedical Sciences, Mayo Clinic, Rochester, MN 55905, United States of America; 4 Department of Physiology and Biomedical Engineering, Mayo Clinic, Rochester, MN 55905, United States of America; 5 Department of Neurology, Mayo Clinic, Rochester, MN 55905, United States of America; 6 Department of Neurologic Surgery, Mayo Clinic, Rochester, MN 55905, United States of America; 7 School of Medicine, Deakin University, Geelong, Victoria 3216, Australia; 8 Division of Experimental Neurology, Mayo Clinic, Rochester, MN 55905, United States of America

**Keywords:** microperfusion, interstitial fluid, EEG, seizure, proteomics

## Abstract

*Objective*. To modify off-the-shelf components to build a device for collecting electroencephalography (EEG) from macroelectrodes surrounded by large fluid access ports sampled by an integrated microperfusion system in order to establish a method for sampling brain interstitial fluid (ISF) at the site of stimulation or seizure activity with no bias for molecular size. *Approach*. Twenty-four 560 *µ*m diameter holes were ablated through the sheath surrounding one platinum–iridium macroelectrode of a standard Spencer depth electrode using a femtosecond UV laser. A syringe pump was converted to push–pull configuration and connected to the fluidics catheter of a commercially available microdialysis system. The fluidics were inserted into the lumen of the modified Spencer electrode with the microdialysis membrane removed, converting the system to open flow microperfusion. Electrical performance and analyte recovery were measured and parameters were systematically altered to improve performance. An optimized device was tested in the pig brain and unbiased quantitative mass spectrometry was used to characterize the perfusate collected from the peri-electrode brain in response to stimulation. *Main results*. Optimized parameters resulted in >70% recovery of 70 kDa dextran from a tissue analog. The optimized device was implanted in the cortex of a pig and perfusate was collected during four 60 min epochs. Following a baseline epoch, the macroelectrode surrounded by microperfusion ports was stimulated at 2 Hz (0.7 mA, 200 *µ*s pulse width). Following a post-stimulation epoch, the cortex near the electrode was stimulated with benzylpenicillin to induce epileptiform activity. Proteomic analysis of the perfusates revealed a unique inflammatory signature induced by electrical stimulation. This signature was not detected in bulk tissue ISF. *Significance*. A modified dual-sensing electrode that permits coincident detection of EEG and ISF at the site of epileptiform neural activity may reveal novel pathogenic mechanisms and therapeutic targets that are otherwise undetectable at the bulk tissue level.

## 1. Introduction

Seizures result in the fluctuation of both neurochemical and electrical biomarkers within the central nervous system (CNS), making simultaneous dual sampling from within the diseased microenvironment valuable for understanding disease mechanisms and treatment modalities [1]. Historic collection techniques including microdialysis [2] and intracranial electroencephalography (EEG) [3, 4] have provided insights into the relationship between neurochemical and electrical fluctuations in the CNS but have been limited with regard to spatial concurrency of the sampling modalities due to the physical separation of each probe type. Most previous designs have also been limited to collection of small molecular weight compounds due to the use of size-restricted semipermeable microdialysis membranes [5], precluding the unbiased collection of large macromolecular complexes and cell-derived membrane-bounded signaling entities such as extracellular vesicles [6–8].

Previous efforts to collect spatially coincident fluid and functional data within the brain include adhesion of microwire electrodes or Teflon-coated stainless steel recording electrodes to the outside of commercially available microdialysis catheters [9, 10] and insertion of wire electrodes into the microdialysis probe lumen [11–13]. The first method is associated with the potential separation of the electrode from the catheter during insertion, resulting in spatial decoherence of the signals, as well as the potential for tissue damage due to the large effective diameter of the electrode-catheter pair. The second method exhibits poor electrophysiological sensitivity due to interference from the microdialysis membrane and signal averaging across the fluid volume inside the probe. A probe design used by Fried and colleagues in which platinum iridium microwires splayed out along the tip of a microdialysis membrane provided unprecedented resolution of changes in neurotransmitter levels associated with single-unit recordings collected during seizures detected by platinum iridium macroelectrodes located along the microdialysis catheter body [14]. Likewise, a modified Spencer electrode with perforations was used by Spencer and colleagues to perform zero-flow microdialysis to measure glutamate release in the hippocampus in response to 50 Hz stimulation [15]. Building on this concept, we sought to use off-the-shelf components to develop a reproducible device for collecting EEG from macroelectrodes surrounded by large fluid access ports sampled by an integrated microperfusion system [1], thereby establishing a method with no bias for molecular or macromolecular size.

## 2. Materials and methods

### 2.1. Electrode modification

**Figure 1.**
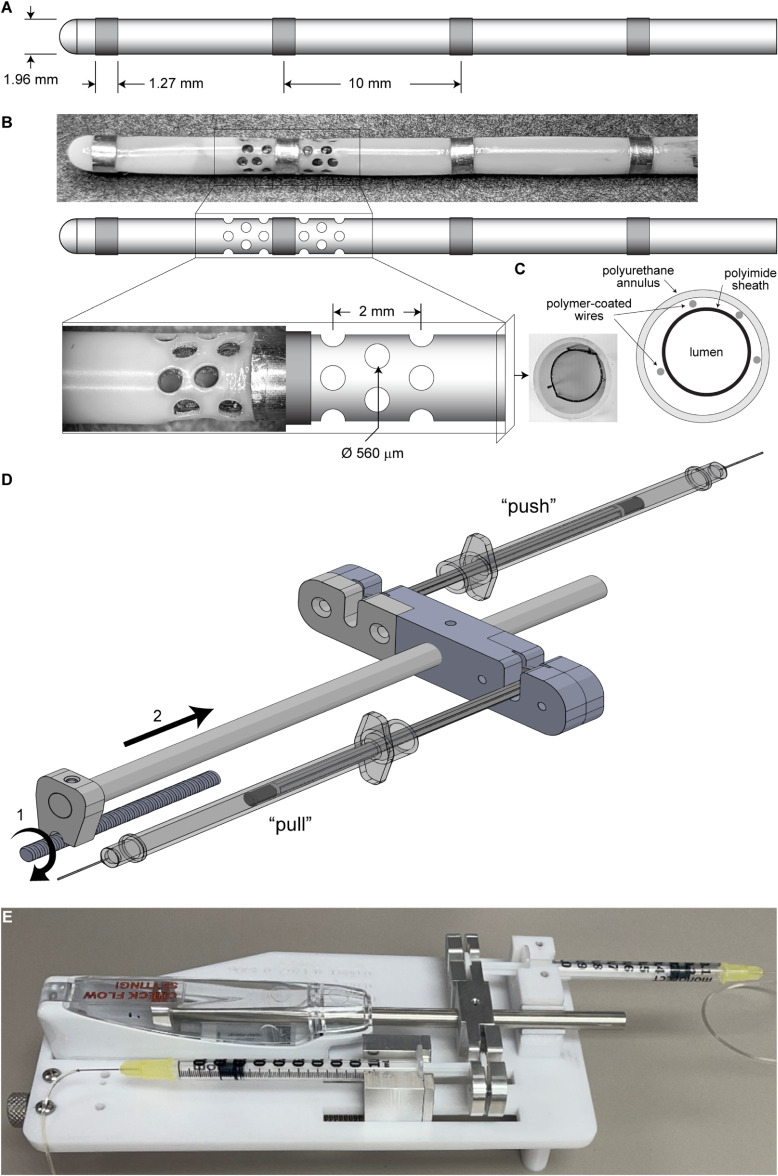
Electrode schematic and modified push-pull pump design. (A) Dimensions of the unmodified Ad-Tech Spencer depth electrode. (B) Location, dimensions, and spatial arrangement of the laser ablated microperfusion access ports in the depth electrode. An example of a modified electrode is shown above the schematic. The boxed region is magnified to reveal port layout. (C) Cross-sectional view through the probe showing the location of the wires, the internal polyimide sheath, and the polyurethane annulus. (D) Schematic of the modification used to convert the rotating lead screw motion of the M Dialysis 107 syringe pump (1) to linear displacement (2) to realize a dual syringe push-pull configuration. (E) Photograph of the modified pump setup with 1 ml syringes attached to the microperfusion fluidics.

The electrode subassembly was a Spencer depth electrode (SD04R-SP10X; AD-TECH) rated for 30 d of implantation, with a maximum charge density of 30 *µ*C cm^−2^ and a safe charge injection capacity of 2.1 *µ*C based on the 0.07 cm^2^ surface area of each macroelectrode. The electrode is constructed as a multi-walled catheter bearing four distal platinum–iridium macroelectrodes connected to proximal contacts by polymer-coated wires running the length of the shaft (figure 1(A)). An internal polyimide sheath separates the lumen of the electrode shaft from the outer polyurethane annulus and serves to protect the wires from the stainless-steel stylus used to stabilize the electrode during insertion (figure 1(C)). In order to permit free exchange of fluid from the surrounding brain tissue into the lumen of the electrode, a triple wavelength femtosecond laser (PhotoMachining Inc) was used to ablate holes through the outer wall and the polyimide sheath. Exposure to femtosecond pulses of light at 343 nm in a cross-hatch pattern permitted ablation without significant heat dissipation [16, 17]. Wires were avoided using transillumination to visualize location and a non-reflective black plastic stylus was inserted into the lumen to prevent scattering and to protect the opposing wall. Ablated material was removed by vacuum during the process. We ablated three rows of four holes per row above and below the second most distal macroelectrode (figure 1(B)). Within a row each hole was positioned 90° apart; the central row of holes was rotated 45° from the top and bottom row. Each row was separated by 1 mm and the row closest to the macroelectrode was positioned 0.5 mm away. Three different focusing strategies were tested during ablation of the outer wall; for the low energy tests, after ablation of the outer wall the laser was focused on the underlying polyimide sheath and ten passes at 35% power was used to remove the material.

### 2.2. Pump and fluidics modification

The microperfusion pump subassembly was a battery-powered 107 syringe pump (M Dialysis AB) with adjustable flow rate (0.1, 0.2, 0.3, 0.5, 1.0, 2.0, 5.0 *µ*l min^−1^). The pump was converted to push–pull operation by incorporating aluminum couplings that concurrently displace the plunger of the push syringe while moving the pull syringe plunger at the identical rate. The lead screw of the original pump was fitted with a threaded coupler that transferred shaft rotation to linear displacement moving each plunger in opposite directions (figure 1(D)). Our modified push–pull pump system used generic 1 ml syringes (figure 1(E)) instead of the proprietary 2.5 ml syringe that comes with the pump, reducing the standard 107 pump flow rates to 0.016, 0.032, 0.048, 0.08, 0.16, 0.32, and 0.80 *µ*l min^−1^.

The fluidics subassembly was from an off-the-shelf M Dialysis-71 high molecular weight (100 kDa) microdialysis catheter with a 20 mm membrane. This membrane was removed, resulting in an open flow inlet/outlet within the lumen of the electrode after insertion. Luer-lock syringe tips (JG28-0.5HPX; Jensen Global) were used to connect the syringes to the microdialysis fluidics.

### 2.3. Electrical impedance testing

Modified electrodes were tested for ablation-induced changes in impedance using an external neurostimulator system delivering a single charge balanced current pulse (0.4 mA, 80 *µ*s pulse width) to determine impedance. Electrodes were tested *in vitro* before and after laser ablation. Each macroelectrode was measured relative to a reference electrode using a saline bath (0.9% NaCl); electrode numbering proceeded proximal-to-distal, with electrode four being the most distal. The modified electrodes were also tested *in vivo* for impedance using the same pulse parameters employed in the *in vitro* tests. In addition to the single frequency test, modified and unmodified electrodes were assessed using electrochemical impedance spectroscopy and a frequency sweep from 1 Hz to 100 kHz, as previously described [18]. Electrodes were tested on a Reference 600 (Gamry Instruments) potentiostat immediately upon insertion into saline containing 0.2 mg ml^−1^ bovine serum albumin. After incubation for 6 h in the solution the electrodes were tested again. Impedance and phase measurements were collected on the third most distal macroelectrode on the probe using a coiled platinum wire counter electrode (Gamry Instruments) referenced to an Ag/AgCl electrode (BASi).

### 2.4. Signal-to-noise ratio (SNR) measurement

The *in vitro* SNR exhibited by modified and unmodified electrodes were measured using artificially generated signal in a saline solution (0.9% NaCl) at 22 °C. Electrodes that had been previously implanted in a pig were cleaned and fixed in parallel in a custom-made plastic reservoir containing 100 ml saline. Background noise on the electrodes was measured in bipolar configuration between the first two macroelectrodes using a Neuralynx-Cube acquisition system. Modified and unmodified electrodes were measured simultaneously for 60 s at 30 kHz using 0.0305 *µ*V quantization steps and bandpass filtering between 0.1 and 7500 Hz. Signal response on the electrodes was tested using an artificial signal mixture comprised of sinewaves of equivalent amplitude at frequencies corresponding to prime numbers between 1 and 41 generated with a National Instruments USB-6251 signal generator and an NPI Electronics ISO-isolation unit. The signal mixture was scaled and injected in current-supply mode with peak-to-peak magnitude of 2 *µ*A into the saline bath using stainless steel electrodes positioned at each end of the reservoir. Signal response was measured for 60 s using the same configuration employed for noise assessment. Voltages detected on the electrodes ranged between 20 and 100 *µ*V, consistent with *in vivo* measurements. The power spectrum density (PSD) in 10 s non-overlapping windows was calculated using MATLAB throughout the noise and stimulated response recordings. The average noise response at each frequency was derived from the mean of all background noise PSD windows. The SNR at each stimulus frequency was calculated using the ratio of the signal response power in each window divided by the average noise power, followed by conversion to dB.

The *in vivo* signal response profile was assessed by calculating the PSD in 4096 frequency bins across 15 kHz in 1 s time windows from 100 s of recording collected shortly after electrode implantation and from 100 s recorded during the end of the 3rd hour of implantation. The mean and standard deviation of the power in the early and late recordings was calculated in each frequency bin. Receiver-operating characteristic curves were calculated for the early and late recordings in Prism and area under the curve (AUC) and standard error were derived. The *z*-score for the difference between curves was calculated by dividing the absolute value of the difference between AUC_late_ and AUC_early_ by the square root of the sum of the squared standard errors for both curves [19]. The *z*-score was used to calculate the two-tailed *P* value against the normal distribution.

### 2.5. Analyte recovery analysis

The no-net-flux method [1, 20, 21] was used to measure recovery of trypan blue, a small molecular weight molecule (870 Da), and fluorescein-conjugated dextran, a large molecular weight molecule (70 kDa). In this method the steady state concentration of analyte in the perfusate (*C*_out_) relative to the input concentration (*C*_in_) is plotted against various input concentrations and the slope of the fitted regression line is used to calculate the percent recovery. Target analytes were either dissolved in saline (0.9% NaCl) or distributed in 0.6% agarose, which served as a tissue surrogate. Analyte recovery was performed at 37 °C and endpoint samples collected after 30 min were measured by absorbance (trypan blue: 580 nm) or fluorescence (dextran: Ex490 nm, Em525 nm).

### 2.6. Pig neurosurgery

All study procedures were performed in accordance with the National Institutes of Health Guidelines for Animal Research (Guide for the Care and Use of Laboratory Animals) and were approved by the Mayo Clinic Institutional Animal Care and Use Committee. Pigs were socially housed in a controlled environment with humidity at 45%, temperature at 21 °C, with once daily feeding and ad libitum access to water. The experimental animal was fasted before the day of surgery. The subject was initially sedated with a mixture of Telazol (tiletamine and zolazepam) (5 mg kg^−1^) and xylazine (2 mg kg^−1^) via intramuscular injection. An endotracheal tube was placed, and an intravenous catheter was inserted and secured in an ear vein. Fentanyl was continuously infused at 10 *µ*g kg^−1^ hr^−1^ through the IV catheter. Anesthesia was maintained with 1.5%–3% isoflurane throughout the surgery. Once the surgical preparation was completed, the pig’s head was fixed in the stereotactic frame. Heart rate, indirect blood pressure, body temperature, and O_2_-saturation were monitored throughout the procedure and recorded every 15 min. When the heart rate stabilized under anesthesia, a midline cutaneous incision was performed in the scalp, burr holes were drilled in the skull, and the dura was opened. The modified microperfusion probe was secured in the Kopf micromanipulator and slowly inserted into the estimated location of the primary sensory cortex through a burr hole. During the 4th collection epoch 2–3 *µ*l of an aqueous solution of benzylpenicillin (1100 U *µ*l^−1^, Penna-G, Sigma) was injected next to the microperfusion probe to induce epileptiform activity [22].

### 2.7. EEG

Electrical signals were collected from the macroelectrodes at 30 kHz using a wireless data acquisition system (Cube-64, NeuraLynx). For analysis, a 400-sample median high-pass filter was applied to remove baseline drift. Line noise was suppressed with a notch filter at 60 Hz and relevant higher harmonics, including 120 and 180 Hz. All data analysis and review were performed in MATLAB R2021 A (MathWorks).

### 2.8. Proteomic analysis

Perfusates were collected into the pull pump fluid line at 0.8 *µ*l min^−1^. At the end of the epoch the perfusate was recovered into a microfuge tube and held on ice prior to freezing at −80 °C. cerebrospinal fluid (CSF) was collected at the electrode implantation site using a blunt needle and syringe. Bulk tissue interstitial fluid (ISF) was prepared using our established method [23], with slight modification. In brief, 10 mm^3^ of cortical tissue was dissected from around the electrode site and placed in ice-cold Hibernate-E media for transport. The tissue was collected by centrifugation at 600 g for 5 min at 4 °C, then resuspended in 300 *µ*l phosphate-buffered saline (PBS) containing 10 *µ*g ml^−1^ aprotinin, 1 *µ*g ml^−1^ leupeptin, and 1 mM phenylmethylsulfonyl fluoride. Following homogenization using ten strokes in a cold glass Tenbroeck homogenizer, the cell suspension was centrifuged at 1000 g for 5 min at 4 °C. The supernatant was collected and clarified by centrifugation at 16 000 g for 5 min at 4 °C.

Total protein levels in the microperfusate, CSF, and tissue ISF were measured and the samples were normalized based on concentration. Samples were processed by drying in a vacuum centrifuge concentrator and digested with 200 ng trypsin/Lys-C (Promega) in 20 mM Tris pH 8.2 containing 0.0005% zwittergent 3-16. Equivalent amounts of the acidified peptide digests were analyzed using a Thermo Scientific Exploris 480 mass spectrometer coupled to a Thermo Ultimate 3000 RSLCnano high performance liquid chromatography (HPLC) system. The digest mixtures were loaded onto a Halo C18 2.7 *µ*m EXP stem trap (Optimize Technologies) and chromatography was performed using 0.1% formic acid in both the A solvent (98% water/2% acetonitrile) and B solvent (80% acetonitrile/10% isopropanol/10% water), with a 3% B to 35% B gradient over 120 min at 350 nl min^−1^ through a PepSep C18 2.4 *µ*m, 100 *µ*m × 40 cm column. The Exploris 480 mass spectrometer was set for data dependent acquisition with a 3 s cycle time between the MS1 survey scan from 340–1600 m z^−1^ at resolution 120 000 (at 200 m z^−1^ ), followed by HCD MS/MS scans at resolution 15 000 with a NCE setting of 30 and the isolation width set to 1.2 m z^−1^. Ions with charge states of 2–4 were allowed for MSMS and if selected, were placed on an exclusion list for 30 s. The normalized AGC target settings were 100% for the MS1 and 80% for MS2 scans with max ion inject times of 50 ms and 120 ms respectively. The raw data were analyzed with MaxQuant (ver 1.6.17.0) using the Andromeda search engine [24] against the NCBI RefSeq pig database downloaded June 2022 and allowing for oxidized methionine and protein N-terminal acetylation as variable modifications. The protein identifications were filtered at a false discovery rate (FDR) [25] of 1% at the peptide and protein level with a two peptide minimum and reported with accompanying intensity-based absolute quantitation (iBAQ) [26] values.

### 2.9. Data analysis and presentation

Except for the *in vivo* microperfusion, experiments were repeated at least twice and at least three replicates were used per condition. Findings are reported as mean ±95% confidence interval where appropriate. Data were analyzed and graphed in Prism (GraphPad). Images were processed in Photoshop (Adobe) and schematics were drawn in Illustrator (Adobe) or Solidworks (Dassault Systèmes). FDRs and enrichment scores in figure 5(B) were calculated using WebGestalt [27]. The heatmap and *Z*-scores in figure 5(C) were calculated using Heatmapper [28].

## 3. Results

### 3.1. Electrode ablation parameters

**Figure 2.**
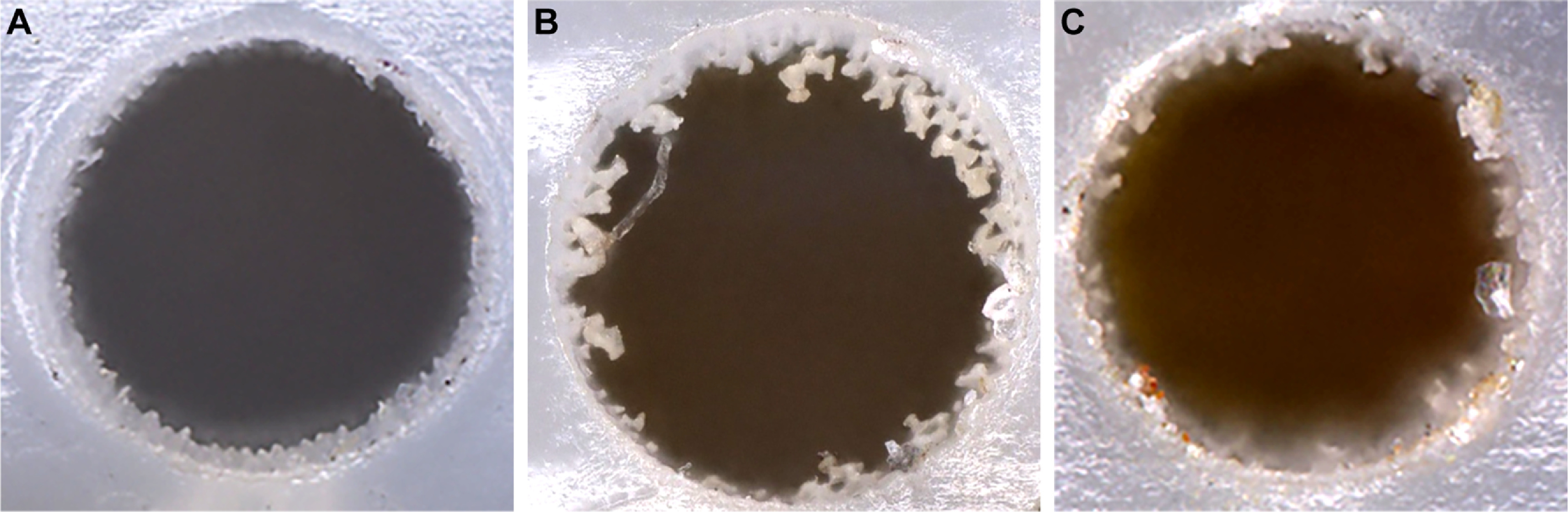
Impact of laser ablation parameters on microperfusion port quality. Femtosecond pulses of 343 nm laser light were used to ablate the polyurethane outer sheath and polyimide inner sheath of the Spencer depth electrode. The ∼20 *µ*m diameter beam (at focus) was fired under different laser powers, beam passes, and focal steps to ablate a 560 *µ*m diameter hole in the electrode. (A) A clean hole was ablated using 50% laser power (1.5 W) and 20 beam passes focused on the exterior surface. However, these parameters damaged the opposing wall. (B) A black plastic stylus was introduced into the lumen to prevent collateral damage and the laser power was reduced to 35% (1.1 W). Using 15 *µ*m pitch with 40 passes and a 5 *µ*m step in focal depth for each pass resulted in incomplete ablation at the perimeter of the hole. (C) Reducing the beam pitch to 10 *µ*m and laser power to 30% while increasing the number of passes to 120 with a 5 *µ*m step in focal depth for each pass resulted in an acceptably clean perimeter without collateral damage or heat dissipation artifacts.

Three approaches were tested to evaluate the collective impact of laser power (100% = 3 W average at 343 nm), focal depth, and number of passes on the quality and efficiency of ablation through both the polyurethane electrode wall and the inner polyimide sheath. In the first, 50% laser power (1.5 W) was focused on the exterior surface of the catheter and 20 passes were made to ablate holes 560 *µ*m in diameter (figure 2(A)). In this approach an internal black plastic stylus was not used and the opposing wall of the internal lumen was partially ablated. In the second approach, the laser power was reduced to 35% (1.1 W) and the focus was stepped radially inward by 5 *µ*m per pass for 40 passes (200 *µ*m of the 600 *µ*m wall thickness). While this protocol successfully ablated both the outer wall and the inner polyimide sheath, a large amount of honeycomb-like material remained around the perimeter of the hole (figure 2(B)). In the third approach, the laser power was reduced to 30% (0.9 W) and the focus was stepped radially inward by 5 *µ*m per pass for 120 passes (through the full 600 *µ*m wall thickness) (figure 2(C)). In addition, the pitch, defined as the spacing between beam paths during the passes, was reduced from 15 *µ*m in the second approach to 10 *µ*m in the third approach, resulting in complete overlap of beam paths. This produced a clean hole without significant collateral damage.

### 3.2. Molecular recovery properties of the modified microperfusion electrode

**Figure 3.**
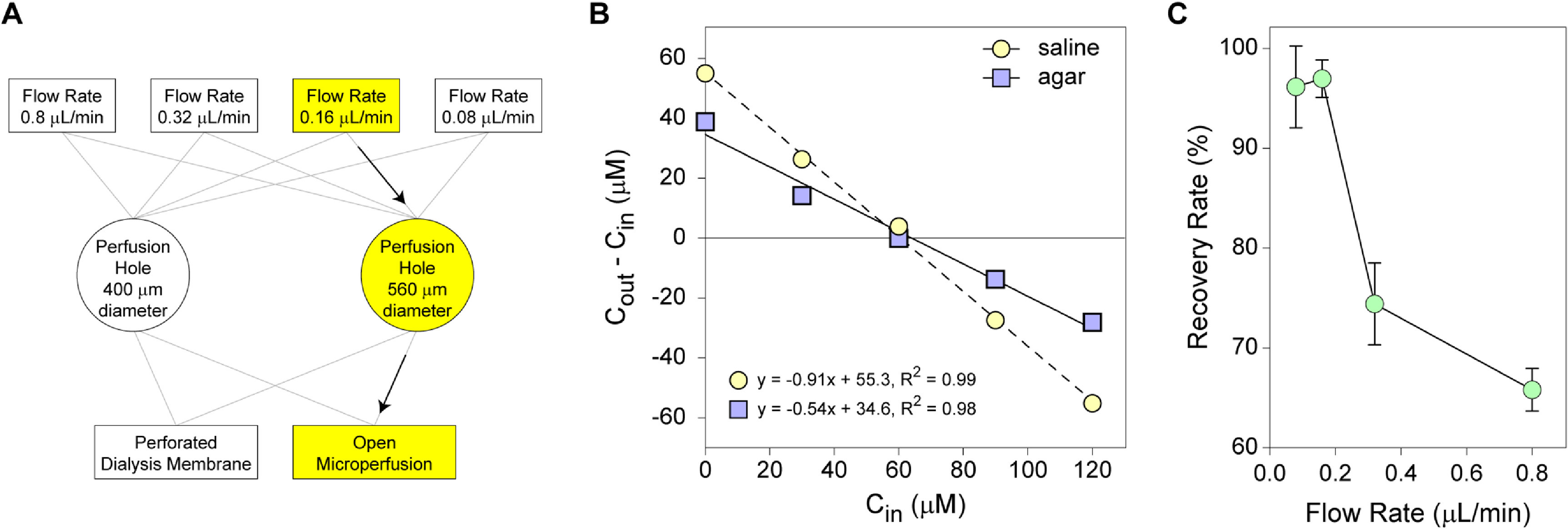
Optimization of device parameters and recovery rate assessment. (A) Fluid flow rate, diameter of the perfusion holes in the depth electrode, and the presence of microdialysis membrane on the fluidics were systematically modified to establish the optimal conditions for analyte recovery (optimal parameters shown in yellow). (B) No-net-flux (NNF) analysis was used to measure recovery of trypan blue dissolved at 60 *µ*M in saline solution (yellow circles) or in a 0.6% agar tissue analog (blue squares). Flow rate was set to 0.8 *µ*l min^−1^ for this assay. The NNF method introduces different concentrations of analyte into the perfusate (*C*_in_) and measures differential recovery in the outflow (*C*_out_–*C*_in_). When the concentration in the inflow equals the actual concentration in the substrate the differential recovery is zero, equivalent to no-net-flux. The recovery when the inflow concentration is zero represents the maximal recovery potential of the system. Fitting a line to the data yields a slope that is equivalent to the effective percent recovery. Trypan blue recovery from saline was 91%; recovery from agar was 54%. Graphs show a representative experiment. (C) Flow rate was adjusted from 0.8 *µ*l min^−1^ down to 0.08 *µ*l min^−1^ and NNF recovery of trypan blue from agar was measured. Recovery plateaued at 97% at 0.16 *µ*l min−1.

As outlined in figure 3(A), a systematic approach was taken to identify the optimal parameters for recovery of analytes using the modified device. In addition to testing the matrix from which the analyte was recovered (saline vs agar), we tested flow rate, perfusion hole size, and presence or absence of perforated microdialysis membrane inside the electrode. We established that recovery of trypan blue using just the modified fluidics was linear from both saline and agar at a fixed flow rate of 0.8 *µ*l min^−1^ in the absence of the electrode (figure 3(B)). Recovery of the small molecular weight analyte from the tissue surrogate was ∼54%. Using this benchmark, a modified calculation was performed using the ratio of analyte in the perfusate (*C*_out_) to the concentration of analyte in the agar (*C*_in_) at various flow rates (figure 3(C)). We found that recovery plateaued at 97% at 0.16 *µ*l min^−1^ and this flow rate was used for the remaining tests. Next, trypan blue recovery from agar was assessed using the complete device setup and the effect of ablated port size was determined. We found that increasing the perfusion hole diameter from 400 *µ*m to 560 *µ*m improved trypan blue recovery from 52.5 ± 4.9% to 61.6 ± 8.6% in the presence of a perforated microdialysis membrane and from 62.8 ± 8.4% to 77.5 ± 12.8% in the absence of membrane (i.e. open microperfusion). Finally, we recovered 72.2 ± 19.8% of 70 kDa dextran from agar using 560 *µ*m ports, no dialysis membrane, and a flow rate of 0.16 *µ*l min^−1^.

### 3.3. Impact of ablation on electrical and mechanical integrity

The integrity of electrical connections in the ablated electrode was assessed by measuring impedance. A Spencer depth electrode as provided by the manufacturer was measured several times before ablation and immediately before and after laser exposure (table 1). Of note, simply testing the electrode for two days after removal from the sterile packaging resulted in a drop in impedance at all four macroelectrodes, despite cleaning and drying the device between measurements. The introduction of laser ablated pores in the electrode resulted in an impedance drop at all four electrodes, not just the 3rd electrode around which the holes were generated (table 1). This suggested that ablating the electrode induced a pervasive change in the electrical nature of the device. We hypothesized that rather than a structural effect, per se, the ablation changed impedance by allowing the free entry of saline from the testing bath into the lumen of the electrode, thereby changing conduction properties of the wire connectors. To test this, we injected saline into an unablated Spencer electrode. This resulted in a ∼29% drop in impedance, which is comparable to the ∼31% drop across all electrodes in the ablated electrode. In parallel, impedance was measured *in vivo* across the two most distal macroelectrodes on modified and unmodified probes using the same parameters. Immediately following implantation, the impedance on the unmodified electrode was 1950 Ω; after 6 h the impedance was 2098 Ω. The modified electrode impedance was 2043 Ω at implantation and 2015 Ω 6 h later.

**Table 1.** The impact of ablation on electrical properties of the Spencer electrode was tested by measuring impedance over time. Each of the four macroelectrodes was measured relative to a reference electrode in a saline bath.

Electrode pair	2 d prior to ablation (Ω)	1 d prior to ablation (Ω)	Immediately preceding ablation (Ω)	Immediately after ablation (Ω)	3 d after ablation (Ω)
Ref/1	1840	1598	1593	793	953
Ref/2	1350	1115	1115	788	913
Ref/3	1998	1433	1433	913	968
Ref/4	1920	1275	1115	788	953

**Figure 4.**
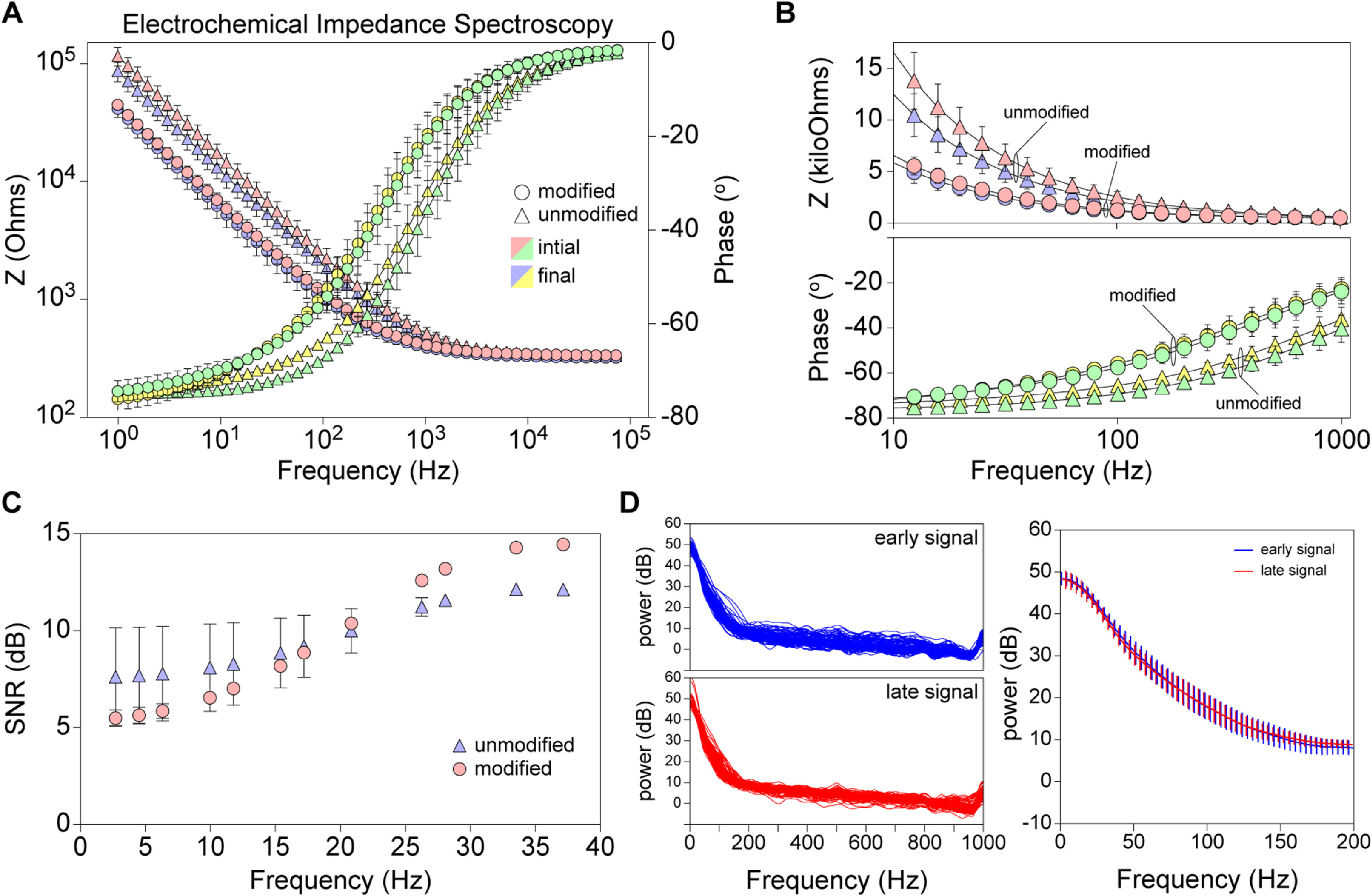
Electrochemical impedance spectroscopy assessment. (A) Bode plot showing the change in impedance (left *y*-axis) and phase (right *y*-axis) across a frequency range of 1 Hz to 100 kHz. The unmodified electrode is represented by a circle; the modified electrode is represented by a triangle. Color coding shows the impedance measured at the start (light red) and end (light blue) of the experiment and the phase measured at the start (light green) and end (light yellow) of the experiment. (B) Impedance and phase data highlighted between 10 and 1000 Hz. The symbol scheme is the same used in A. (C) Signal-to-noise response profile of explanted modified (red circle) and unmodified (blue triangle) electrodes measured in response to mixed signal 2 *µ*A peak-to-peak current injection for 60 s at 30 kHz in a saline bath. Stimulus responses were assessed at prime number frequencies between 1 and 41 Hz. Graph shows mean ± standard deviation of six 10 s epochs at each frequency. (D) *In vivo* signal power from 100 s of recording collected immediately after implantation (‘early’) and from 100 s recorded at the end of the 3rd hour of recording (‘late’). Traces on the left show the power from 0.1 to 1000 Hz in 100 one-second epochs per recording time. Graph on the right shows the average power from 0.1 to 200 Hz across the 100 s recording epochs, with standard deviation shown for each of the frequency bins. Early signal is shown in blue; late signal is shown in red.

Unmodified and modified electrodes were also assessed by electrochemical impedance spectroscopy for changes across a frequency spectrum (1 Hz–100 kHz) (figure 4). Impedance and phase were measured at the time of insertion into a saline bath containing protein (bovine serum albumin) and again after incubation for 6 h in the solution, to model the effects of implantation. The Bode plot (figure 4(A)) indicates that the modified electrode impedance and phase across the frequency sweep at outset was different than the unmodified electrode characteristics. However, across the 10–1000 Hz range that is most relevant to EEG, the modified electrode was comparable to the unmodified device (figure 4(B)). Two-way ANOVA detected no significant differences in impedance at any frequencies (*F*_(150 200)_ = 13.12, *P* < 0.0001; Tukey’s multiple comparison test between electrodes at each frequency) and the electrode modification only accounted for 4% of the total variance in impedance. Critically, there was no difference at any frequency in the impedance measured on the modified electrode at the end of the incubation relative to the start.

Signal-to-noise performance of unmodified and modified electrodes was assessed *in vitro* following explantation from the pig brain (figure 4(C)). Discrete responses at stimulation frequencies between 1 and 41 Hz were measured in 10 s epochs and the SNR was determined relative to the background noise. The overall SNR was not impacted by introduction of macroscopic perfusion ports in the modified electrode. Likewise, analysis of the power spectrum of the signal recorded from the modified electrode immediately after implantation into the pig brain versus the power of the signal 3 h later revealed no significant difference in the electrode response (*P* = 0.6360 between frequency response curves), indicating that the performance of the electrode was not degraded by active and ongoing microperfusion across the macropores (figure 4(D)).

Mechanical integrity was measured using a force gauge positioned coaxially against the probe tip. Buckling strength of the modified electrode was 1.0 N, compared to 3.8 N for the unmodified probe. The reported maximum penetration transient for 0.6% agarose or pig brain is <0.1 N with a continuing penetration force less than 0.03 N [29]. In addition, the electrode is implanted during *in vivo* experiments with an internal stylet inserted in the lumen. Once implanted, the stylet is removed and the microperfusion subassembly is inserted. Overall, these findings indicate that ablation does not compromise the mechanical integrity of the probe to an extent that impacts surgical implantation.

### 3.4. Unbiased proteomic analysis of stimulation-induced changes in cortical microperfusate

**Figure 5.**
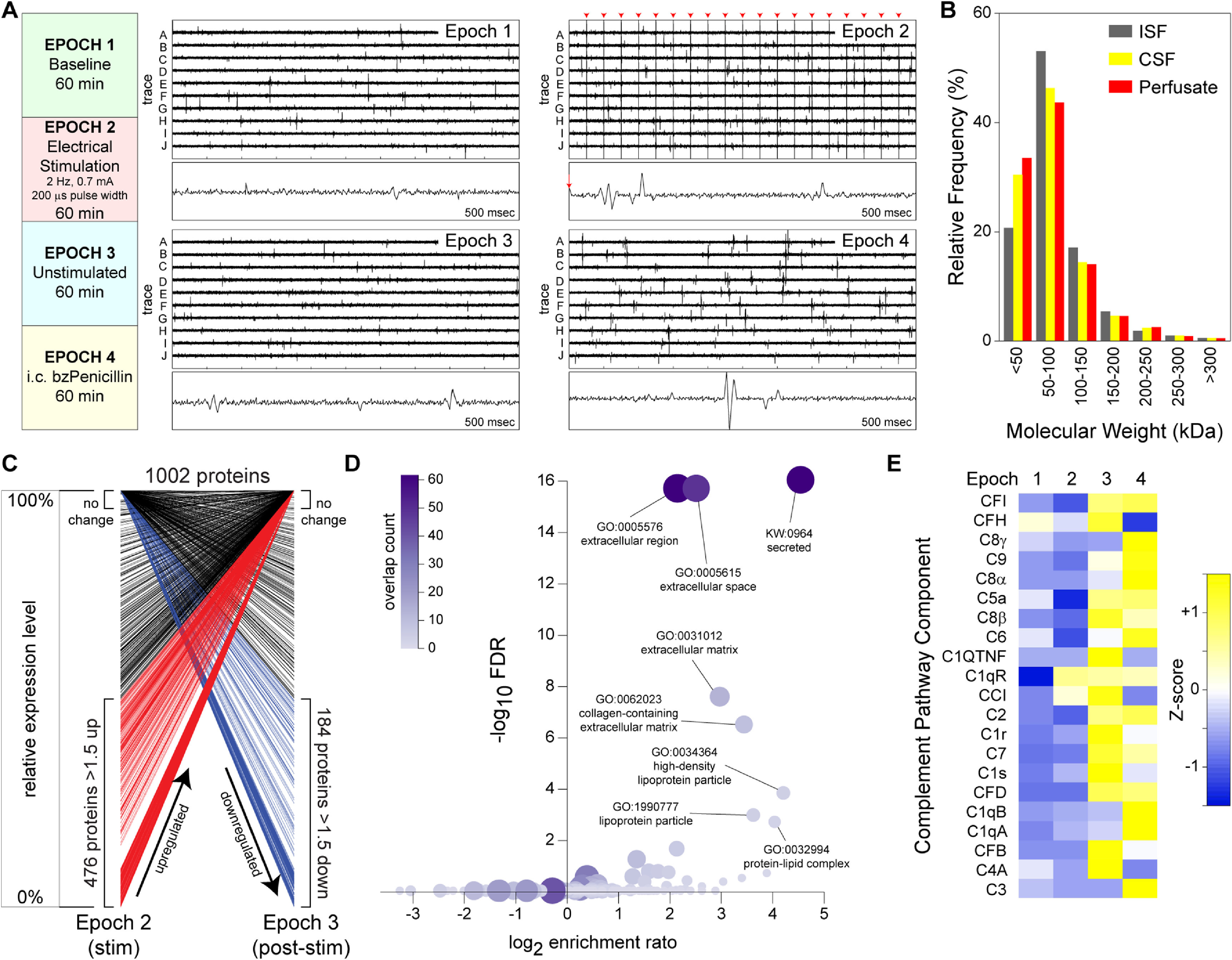
*In vivo* microperfusate collection and proteomic analysis. (A) A modified microperfusion-EEG electrode was placed in the cortex of a pig and perfusate and coincident EEG were collected during 4 one-hour epochs. Ten representative 10 s EEG traces collected on the macroelectrode bordered by perfusion holes are shown for each epoch; a single representative 500 msec trace is also shown. Note that the large repetitive signals in epoch 2 correspond with the 2 Hz electrical stimulation driven on the recording macroelectrode (red arrow heads); the 500 msec trace shows the EEG immediately after a 0.7 mA, 200 *µ*s pulse. (B) Perfusate from each epoch (combined analysis of all four samples; red), total tissue ISF (gray), and CSF (yellow) were analyzed using quantitative mass spectrometry and the relative frequency of detectable proteins in 50 kDa bins is shown. (C) Of 1002 proteins detected in either epoch 2 or epoch 3, 476 were upregulated more than 1.5-fold following stimulation (red lines) and 184 were downregulated more than 1.5-fold (blue lines). The graph shows the normalized percent expression between epochs, with many of the differentially regulated proteins either changing from undetectable to detectable (bottom left to upper right) or from detectable to undetectable (top left to bottom right). Overall, electrical stimulation led to a robust change in over 65% of the proteins detected in the perfusate. (D) Over-representation analysis of the proteins increased more than 1.5-fold following electrical stimulation (i.e. the red lines in panel C) indicated that secreted extracellular proteins were significantly enriched. The enrichment score was calculated based on the number of upregulated proteins overlapping the membership of each gene ontology cluster (color-coded in purple). The false discovery rate (FDR) was calculated based on expected stochastic overlap. (E) Twenty one complement pathway proteins were detected in the perfusate from at least one epoch. The heat map shows *z*-scores calculated for each complement factor across the 4 epochs. Nearly all detectable complement proteins were increased in epoch 3 or 4 or both relative to the first two epochs, suggesting profound upregulation of the complement pathway by electrical stimulation.

The parameters established above were used to collect perfusate from porcine cortex during four ∼60 min epochs (figure 5(A)). Epoch 1 captured baseline proteins and macromolecular complexes (289 ng *µ*l^−1^ protein; 40 *µ*l). Epoch 2 collected perfusate while the macroelectrode surrounded by ablated perfusion holes was electrically stimulated at 2 Hz (0.7 mA, 200 *µ*s pulse width) (300 ng *µ*l^−1^ protein; 40 *µ*l). Epoch 3 collected post-stimulation perfusate (287 ng *µ*l^−1^ protein; 45 *µ*l). Epoch 4 collected fluid while the neighboring cortex was stimulated by injection of benzylpenicillin (236 ng *µ*l^−1^ protein; 70 *µ*l). After the final perfusate epoch, CSF was collected (101 ng *µ*l^−1^ protein; 150 *µ*l) and a tissue block from the site of electrode implantation was dissected and processed for ISF isolation (1.18 *µ*g *µ*l^−1^ protein; 1.75 ml). Mass spectrometry with intensity-based absolute quantification [26] was performed using 3 *µ*g protein from each perfusate sample and from CSF and ISF. Notably, in contrast to the loss of large molecular weight proteins normally associated with microdialysis, using microperfusion we readily recovered the same distribution of proteins by size in the perfusate as observed in CSF and ISF, including 248 proteins larger than 100 kDa (figure 5(B)).

Intensity-based absolute quantification using a FDR of 1% and a minimum of at least two unique peptides from the expected tryptic library to qualify as ‘detected’ resulted in identification of 784 proteins in epoch 1, 756 proteins in epoch 2, 850 proteins in epoch 3, 689 proteins in epoch 4, 956 proteins in CSF, and 2855 proteins in the tissue ISF. In addition, 219 proteins were found only in the perfusates and not in the CSF, while 173 proteins were found only in the CSF and not in the perfusates. Likewise, 550 proteins were detected in the perfusates that were not found in the ISF. Finally, 154 proteins were unique to the perfusates and were not detected in either CSF or ISF. Within the perfusate samples, four response profiles were assessed: (a) proteins that were undetected at baseline and became detectable after stimulation; (b) proteins that were detectable at baseline but were increased >1.5-fold after stimulation; (c) proteins that were detected at baseline but become undetectable after stimulation; (d) proteins that were decreased >1.5-fold after stimulation but were still detectable. As shown in table 2 and figure 5(C), we found 180 category 1 proteins, 296 category 2 proteins, 99 category 3 proteins, and 85 category 4 proteins. Gene ontology profiling of category 1 and 2 proteins using over-representation analysis in WebGestalt [27] revealed a robust enrichment in extracellular proteins, matrix proteins, and lipoproteins (figure 5(D)), consistent with the expectation that most factors collected in the perfusate would be soluble proteins. Indeed, of the category 1/2 proteins, 141 are characterized as secreted in the UniProtKB cellular component keyword hierarchy [30].

**Table 2.** Proteins increased or decreased in perfusate after stimulation. Category 1 = proteins that were undetected at baseline and became detectable after stimulation. Category 2 = proteins that were detectable at baseline but were increased >150% after stimulation. Category 3 = proteins that were detected at baseline but become undetectable after stimulation. Category 4 = proteins that were decreased >150% after stimulation but were still detectable.

Category	GenInfo ID	Symbol	Protein
1	GI|1191864745	ACHE	Acetylcholinesterase
1	GI|753704404	ADA	Adenosine deaminase
1	GI|20177830	ADA2	Adenosine deaminase 2
1	GI|753704402	ADAM10	ADAM metallopeptidase domain 10
1	GI|1191844482	ADAM23	ADAM metallopeptidase domain 23
1	GI|1191873501	ADAMTS4	ADAM metallopeptidase with thrombospondin type 1 motif 4
1	GI|1191901946	ADGRL3	Adhesion G protein-coupled receptor L3
1	GI|753704390	ADPGK	ADP dependent glucokinase
1	GI|399038	ANG	Angiogenin
1	GI|255683368	ANXA1	Annexin A1
1	GI|1191818753	AP2B1	Adaptor related protein complex 2 subunit beta 1
1	GI|1191892934	APLP1	Amyloid beta precursor like protein 1
1	GI|1191860111	ARSB	Arylsulfatase B
1	GI|1191835354	ASAH2	N-acylsphingosine amidohydrolase 2
1	GI|311276152	ATP6AP2	ATPase H+ transporting accessory protein 2
1	GI|347667058	BCHE	Butyrylcholinesterase
1	GI|1191856375	C1QTNF4	C1q and TNF related 4
1	GI|1191910537	C4BPA	Complement component 4 binding protein alpha
1	GI|86604379	C8B	Complement C8 beta chain
1	GI|1191891457	CACHD1	Cache domain containing 1
1	GI|545812853	CAMK2A	Calcium/calmodulin dependent protein kinase II alpha
1	GI|1191819714	CANT1	Calcium activated nucleotidase 1
1	GI|1191840769	CAPN10	Calpain 10
1	GI|1191854271	CAST	Calpastatin
1	GI|927140451	CD177	CD177 molecule
1	GI|1191829651	CD200	CD200 molecule
1	GI|1191808524	CDH7	Cadherin 7
1	GI|1191884956	CDH8	Cadherin 8
1	GI|311271452	CDHR1	Cadherin related family member 1
1	GI|643471	CHI3L1	Chitinase 3 like 1
1	GI|1191828556	CHRD	Chordin
1	GI|1191892825	CNTNAP4	Contactin associated protein family member 4
1	GI|545820332	COL14A1	Collagen type XIV alpha 1 chain
1	GI|1191810736	COL15A1	Collagen type XV alpha 1 chain
1	GI|1191872119	CTHRC1	Collagen triple helix repeat containing 1
1	GI|311263676	CTSC	Cathepsin C
1	GI|1191907786	DCHS1	Dachsous cadherin-related 1
1	GI|1191894907	DDR1	Discoidin domain receptor tyrosine kinase 1
1	GI|18203301	DLST	Dihydrolipoamide S-succinyltransferase
1	GI|298104097	DNAJC3	DnaJ heat shock protein family
1	GI|1191870651	EFNA1	Ephrin A1
1	GI|545798614	EPHA7	EPH receptor A7
1	GI|545849096	ESAM	Endothelial cell adhesion molecule
1	GI|1191863853	FAM20C	FAM20C golgi associated secretory pathway kinase
1	GI|1191845812	FAT2	FAT atypical cadherin 2
1	GI|545815707	FBLN7	Fibulin 7
1	GI|3211746	FOLH1B	Folate hydrolase 1B
1	GI|1191903840	FSTL5	Follistatin like 5
1	GI|18028088	GALNS	Galactosamine
1	GI|1191890799	GALNT1	Polypeptide N-acetylgalactosaminyltransferase 1
1	GI|417515619	GALNT10	Polypeptide N-acetylgalactosaminyltransferase 10
1	GI|1191834685	GALNT7	Polypeptide N-acetylgalactosaminyltransferase 7
1	GI|937575768	GAS6	Growth arrest specific 6
1	GI|12802914	GDF11	Growth differentiation factor 11
1	GI|1191840014	GGT5	Gamma-glutamyltransferase 5
1	GI|1191803922	GMFB	Glia Maturation factor beta
1	GI|927109126	GNPTG	N-acetylglucosamine-1-phosphate transferase subunit gamma
1	GI|1191879911	GNS	Glucosamine
1	GI|1191895933	GPLD1	Glycosylphosphatidylinositol specific phospholipase D1
1	GI|1191900027	H2B1	Histone H2B type 1
1	GI|1191900033	H2B1H	Histone H2B type 1-H-like
1	GI|1191900035	H2B1K	Histone H2B type 1-K
1	GI|1191896036	H2B1M	Histone H2B type 1-M
1	GI|1191874626	H2B2F	Histone H2B type 2-F
1	GI|1191900011	H2BC4	Histone H2B type 1-C4
1	GI|178056464	HEXA	Hexosaminidase subunit alpha
1	GI|343887370	HIST1H2BD	Histone cluster 1, H2bd
1	GI|545812437	HSP70	Heat shock protein family A
1	GI|47522774	HSP90AA1	Heat shock protein 90 alpha family class A member 1
1	GI|1191808723	IGDCC4	Immunoglobulin superfamily DCC subclass member 4
1	GI|78100178	IGFBP1	Insulin like growth factor binding protein 1
1	GI|152937561	IGFBP6	Insulin like growth factor binding protein 6
1	GI|1191915532	IGSF1	Immunoglobulin superfamily member 1
1	GI|545865280	IL1RAP	Interleukin 1 receptor accessory protein
1	GI|389618957	IL6	Interleukin 6
1	GI|83026499	ITM2B	Integral membrane protein 2B
1	GI|1191901140	KIF13A	Kinesin family member 13A
1	GI|1191834581	KIF13B	Kinesin family member 13B
1	GI|1191890289	LAMA1	Laminin subunit alpha 1
1	GI|190360655	LBP	Lipopolysaccharide binding protein
1	GI|54888450	LOC100038329	Immunoglobulin heavy variable 3-23
1	GI|119662961	LOC100125542	Immunoglobulin heavy variable 3-23
1	GI|1191895990	LOC100152135	Histone H2B type 1-A-like
1	GI|1191895988	LOC100152878	Histone H2B type 1-A
1	GI|194038353	LOC100156325	Serpin A3-6
1	GI|1191900031	LOC100156932	Histone H2B type 1
1	GI|194039814	LOC100156937	Histone H2B type 1
1	GI|1191900037	LOC100512420	Histone H2B type 1-K
1	GI|1191900013	LOC100621915	Histone H2B type 1-N
1	GI|23598143	LOC100622791	BOLA class I histocompatibility antigen, alpha chain BL3-7
1	GI|1191836580	LOC100623670	Uncharacterized LOC100623670
1	GI|350591165	LOC100736951	15 kDa protein B-like
1	GI|1191802196	LOC110258825	Uncharacterized LOC110258825
1	GI|335299353	LRRN1	Leucine rich repeat neuronal 1
1	GI|1191825125	LTF	Lactotransferrin
1	GI|1143948	LYZ	Lysozyme
1	GI|1191862665	MAN2A1	Mannosidase alpha class 2A member 1
1	GI|545865216	MASP1	MBL associated serine protease 1
1	GI|290048156	MGP	Matrix Gla protein
1	GI|194043083	MLEC	Malectin
1	GI|311263813	MMP8	Matrix metallopeptidase 8
1	GI|83921637	MMP9	Matrix metallopeptidase 9
1	GI|178057323	MOG	Myelin oligodendrocyte glycoprotein
1	GI|1191825585	MST1	Macrophage stimulating 1
1	GI|350585502	MXRA8	Matrix remodeling associated 8
1	GI|1191906174	NECTIN1	Nectin cell adhesion molecule 1
1	GI|56606059	NPPC	Natriuretic peptide C
1	GI|1191909839	NRGN	Neurogranin
1	GI|1191804145	NTRK3	Neurotrophic receptor tyrosine kinase 3
1	GI|1191856958	NUCB2	Nucleobindin 2
1	GI|123299969	OLFML2A	Olfactomedin like 2A
1	GI|545823604	OLFML3	Olfactomedin like 3
1	GI|1191904668	PCDH10	Protocadherin 10
1	GI|1191817563	PCDH17	Protocadherin 17
1	GI|347300200	PCDH7	Protocadherin 7
1	GI|1191816758	PCDH9	Protocadherin 9
1	GI|1191831192	PCP4	Purkinje cell protein 4
1	GI|1191846478	PDE4D	Phosphodiesterase 4D
1	GI|1191847699	PDYN	Prodynorphin
1	GI|417515730	PFKP	Phosphofructokinase, platelet
1	GI|1191888408	PGD	Phosphogluconate dehydrogenase
1	GI|47575891	PGLYRP1	Peptidoglycan recognition protein 1
1	GI|6647580	PGRMC1	Progesterone receptor membrane component 1
1	GI|343488483	PI16	Peptidase inhibitor 16
1	GI|1018191625	PLA2G7	Phospholipase A2 group VII
1	GI|1191834723	PLBD2	Phospholipase B domain containing 2
1	GI|17467356	PLTP	Phospholipid transfer protein
1	GI|1191827215	PODXL2	Podocalyxin like 2
1	GI|118403870	POMGNT1	Protein O-linked mannose N-acetylglucosaminyltransferase 1
1	GI|545854578	POSTN	Periostin
1	GI|927108570	PPL	Periplakin
1	GI|1191890103	PPT1	Palmitoyl-protein thioesterase 1
1	GI|1191811720	PRRC2B	Proline rich coiled-coil 2B
1	GI|927104676	PRTN3	Proteinase 3
1	GI|545870464	PSAP	Prosaposin
1	GI|1191812339	PTPRK	Protein tyrosine phosphatase receptor type K
1	GI|1191907131	PUS7	Pseudouridine synthase 7
1	GI|1191868583	QPCT	Glutaminyl-peptide cyclotransferase
1	GI|89143155	RETN	Resistin
1	GI|927201117	RNF13	Ring finger protein 13
1	GI|9857227	RPN1	Ribophorin I
1	GI|113205682	S100A6	S100 calcium binding protein A6
1	GI|195969551	S100A8	S100 calcium binding protein A8
1	GI|226442051	S100A9	S100 calcium binding protein A9
1	GI|113205658	SAA3	Serum amyloid A-3 protein
1	GI|1191892995	SBSN	Suprabasin
1	GI|198282079	SELENOP	Selenoprotein P
1	GI|927105772	SEMA6A	Semaphorin 6A
1	GI|1191807574	SEMA6D	Semaphorin 6D
1	GI|194037165	SEPTIN3	Septin 3
1	GI|194038342	SERPINA5	Serpin family A member 5
1	GI|20977253	SERPINA6	Serpin family A member 6
1	GI|456753166	SERPINB1	Serpin family B member 1
1	GI|1191899558	SETD3	SET domain containing 3, actin histidine methyltransferase
1	GI|1191916059	SH3BGRL	SH3 domain binding glutamate rich protein like
1	GI|610497438	SLA-2	MHC class I antigen 2
1	GI|1191819606	SLC38A10	Solute carrier family 38 member 10
1	GI|1191843680	SLC39A10	Solute carrier family 39 member 10
1	GI|217031226	SLC9A3R1	SLC9A3 regulator 1
1	GI|311277072	SLITRK4	SLIT and NTRK like family member 4
1	GI|1228060	SPAI-2	Sodium/potassium ATPase inhibitor SPAI-2
1	GI|350581117	SPOCK1	Sparc/osteonectin, cwcv and kazal-like domains proteoglycan (testican) 1
1	GI|545846762	SPP1	Secreted phosphoprotein 1
1	GI|47523280	SPP2	Secreted phosphoprotein 2
1	GI|311270685	SPRING1	SREBF pathway regulator in golgi 1
1	GI|753703048	SRGN	Serglycin
1	GI|311252046	ST6GAL2	ST6 beta-galactoside alpha-2,6-sialyltransferase 2
1	GI|57528035	STMN1	Stathmin 1
1	GI|1191832492	SUSD5	Sushi domain containing 5
1	GI|1964	TCN1	Transcobalamin 1
1	GI|1191820470	TEX2	Testis expressed 2
1	GI|347300340	THBS1	Thrombospondin 1
1	GI|1191834390	TMEM132D	Transmembrane protein 132D
1	GI|1191895815	TNXB	Tenascin XB
1	GI|753702938	TSC1	TSC complex subunit 1
1	GI|311251027	TSC22D4	TSC22 domain family protein 4
1	GI|315506985	VLDLR	Very low density lipoprotein receptor
1	GI|1191912440	VSIG4	V-set and immunoglobulin domain containing 4
1	GI|545833220	VWA1	Von Willebrand factor A domain containing 1
1	GI|330864705	WAP-3	Elafin family member
2	GI|1146187804	ACE	Angiotensin I converting enzyme
2	GI|1191910611	ADAM22	ADAM metallopeptidase domain 22
2	GI|311272336	ADAM9	ADAM metallopeptidase domain 9
2	GI|1191840007	ADAMDEC1	ADAM like decysin 1
2	GI|1191858051	ADGRL1	Adhesion G protein-coupled receptor L1
2	GI|90903447	AEBP1	AE binding protein 1
2	GI|1191842006	AGA	Aspartylglucosaminidase
2	GI|1191888029	AGRN	Agrin
2	GI|1191839081	AGT	Angiotensinogen
2	GI|545865183	AHSG	Alpha 2-HS glycoprotein
2	GI|927202698	ALCAM	Activated leukocyte cell adhesion molecule
2	GI|345441771	ALDOC	Aldolase, fructose-bisphosphate C
2	GI|1191894245	ALPL	Alkaline phosphatase, biomineralization associated
2	GI|1191871250	AMY2	Amylase, alpha 2B
2	GI|1191892936	APLP1	Amyloid beta precursor like protein 1
2	GI|1191910079	APLP2	Amyloid beta precursor like protein 2
2	GI|164359	APOA1	Apolipoprotein A1
2	GI|3913046	APOA4	Apolipoprotein A4
2	GI|164361	APOC3	Apolipoprotein C3
2	GI|1191828901	APOD	Apolipoprotein D
2	GI|47523674	APOE	Apolipoprotein E
2	GI|1191825140	APP	Amyloid beta precursor protein
2	GI|47522624	ARSA	Arylsulfatase A
2	GI|350594565	ASAH1	N-acylsphingosine amidohydrolase 1
2	GI|1191916177	ATP6AP1	ATPase H+ transporting accessory protein 1
2	GI|927111804	ATP6V1E2	ATPase H+ transporting V1 subunit E2
2	GI|183223979	AXL	AXL receptor tyrosine kinase
2	GI|1191807597	B2M	Beta-2-microglobulin
2	GI|403330337	B4GAT1	Beta-1,4-glucuronyltransferase 1
2	GI|350583290	BCAN	Brevican
2	GI|343790848	BPNT2	3ʹ(2ʹ), 5ʹ-bisphosphate nucleotidase 2
2	GI|166244451	BSG	Basigin
2	GI|545860946	BTD	Biotinidase
2	GI|38455782	C1QA	Complement C1q A chain
2	GI|38455784	C1QC	Complement C1q C chain
2	GI|38455776	C1R	Complement C1r
2	GI|1191877805	C1S	Complement C1s
2	GI|156120138	C2	Complement C2
2	GI|335284688	C3H16orf89	Chromosome 3 C16orf89 homolog
2	GI|178056221	C4A	Complement C4A
2	GI|148226535	C6	Complement C6
2	GI|5639969	C7	Complement C7
2	GI|1134928673	C8A	Complement C8 alpha chain
2	GI|355390262	C8B	Complement C8 beta chain
2	GI|148233690	C9	Complement C9
2	GI|1191909290	CADM1	Cell adhesion molecule 1
2	GI|1191873715	CADM3	Cell adhesion molecule 3
2	GI|387912908	CALR	Calreticulin
2	GI|223469450	CBLN1	Cerebellin 1 precursor
2	GI|1191885857	CCER2	Coiled-coil glutamate rich protein 2
2	GI|350578417	CD109	CD109 molecule
2	GI|117168952	CD14	CD14 molecule
2	GI|1191915579	CD99L2	CD99 molecule like 2
2	GI|209875157	CDH1	Cadherin 1
2	GI|158262677	CDH13	Cadherin 13
2	GI|1191890618	CDH2	Cadherin 2
2	GI|48976065	CDH5	Cadherin 5
2	GI|1191847015	CDH6	Cadherin 6
2	GI|148724909	CFB	Complement factor B
2	GI|927104678	CFD	Complement factor D
2	GI|47523636	CFH	Complement factor H
2	GI|49066631	CHGB	Chromogranin B
2	GI|1191826734	CHL1	Cell adhesion molecule L1 like
2	GI|927219588	CHRDL1	Chordin like 1
2	GI|1191881035	CLSTN3	Calsyntenin 3
2	GI|1191833790	CLU	Clusterin
2	GI|350589462	CNTFR	Ciliary neurotrophic factor receptor
2	GI|350584500	CNTN1	Contactin 1
2	GI|1191910406	CNTN2	Contactin 2
2	GI|1191831064	CNTN4	Contactin 4
2	GI|1191875281	COL11A1	Collagen type XI alpha 1 chain
2	GI|1191832454	COL18A1	Collagen type XVIII alpha 1 chain
2	GI|1191821606	COL1A1	Collagen type I alpha 1 chain
2	GI|1159729723	COL1A2	Collagen type I alpha 2 chain
2	GI|62461592	COL5A1	Collagen type V alpha 1 chain
2	GI|1191843512	COL6A3	Collagen type VI alpha 3 chain
2	GI|1191890496	COLEC12	Collectin subfamily member 12
2	GI|194040626	CPB2	Carboxypeptidase B2
2	GI|121488663	CPE	Carboxypeptidase E
2	GI|343887438	CPQ	Carboxypeptidase Q
2	GI|1191873349	CREG1	Cellular repressor of E1A stimulated genes 1
2	GI|311260574	CRISP3	Cysteine-rich secretory protein 3
2	GI|335284508	CRYM	Crystallin mu
2	GI|35208827	CSF1	Colony stimulating factor 1
2	GI|1191825502	CSPG5	Chondroitin sulfate proteoglycan 5
2	GI|110006616	CST3	Cystatin C
2	GI|335281483	CST6	Cystatin E/M
2	GI|187470655	CTSB	Cathepsin B
2	GI|83523775	CTSD	Cathepsin D
2	GI|545854185	CTSV	Cathepsin V
2	GI|1191825538	DAG1	Dystroglycan 1
2	GI|1191812622	DCC	DCC netrin 1 receptor
2	GI|88606665	DKK3	Dickkopf WNT signaling pathway inhibitor 3
2	GI|1191799745	DPP7	Dipeptidyl peptidase 7
2	GI|1191890625	DSC2	Desmocollin 2
2	GI|1191874572	ECM1	Extracellular matrix protein 1
2	GI|927109316	ECM2	Extracellular matrix protein 2
2	GI|927151561	EFCAB14	EF-hand calcium binding domain 14
2	GI|927111392	EFEMP1	EGF containing fibulin extracellular matrix protein 1
2	GI|545806308	EFEMP2	EGF containing fibulin extracellular matrix protein 2
2	GI|157326747	EFNB2	Ephrin B2
2	GI|311253447	ENPP2	Ectonucleotide pyrophosphatase/phosphodiesterase 2
2	GI|194039425	ENPP5	Ectonucleotide pyrophosphatase/phosphodiesterase family member 5
2	GI|75039077	F12	Coagulation factor XII
2	GI|106647506	F2	Coagulation factor II, thrombin
2	GI|18202922	F5	Coagulation factor V
2	GI|1191912689	F9	Coagulation factor IX
2	GI|311275542	FAM3C	FAM3 metabolism regulating signaling molecule C
2	GI|545824398	FBLN1	Fibulin 1
2	GI|1191827067	FBLN2	Fibulin 2
2	GI|1191899385	FBLN5	Fibulin 5
2	GI|5739075	FBN1	Fibrillin 1
2	GI|52346216	FGL2	Fibrinogen like 2
2	GI|1191847743	FKBP1A	FKBP prolyl isomerase 1A
2	GI|6606280	FMOD	Fibromodulin
2	GI|927173361	FOLR3	Folate receptor 3
2	GI|1191829441	FSTL1	Follistatin like 1
2	GI|1191812215	FUCA2	Alpha-L-fucosidase 2
2	GI|346716243	GAP43	Growth associated protein 43
2	GI|89573945	GAPDH	Glyceraldehyde-3-phosphate dehydrogenase
2	GI|1191847183	GM2A	Ganglioside GM2 activator
2	GI|147223415	GNAS	GNAS complex locus
2	GI|545864627	GOLIM4	Golgi integral membrane protein 4
2	GI|1191814816	GOLM1	Golgi membrane protein 1
2	GI|545876399	GPC1	Glypican 1
2	GI|335305392	GPR37	G protein-coupled receptor 37
2	GI|113205762	GRN	Granulin precursor
2	GI|121118	GSN	Gelsolin
2	GI|1191842403	HERC2	HECT and RLD domain containing E3 ubiquitin protein ligase 2
2	GI|1149122746	HEXB	Hexosaminidase subunit beta
2	GI|350587301	HGFAC	HGF activator
2	GI|541628	HPX	Hemopexin
2	GI|1191888849	HSPG2	Heparan sulfate proteoglycan 2
2	GI|92111118	IGFALS	Insulin like growth factor binding protein acid labile subunit
2	GI|78100179	IGFBP2	Insulin like growth factor binding protein 2
2	GI|84663826	IGFBP5	Insulin like growth factor binding protein 5
2	GI|1111817886	IGFBP7	Insulin like growth factor binding protein 7
2	GI|753703710	IGSF8	Immunoglobulin superfamily member 8
2	GI|1191845399	IL6ST	Interleukin 6 cytokine family signal transducer
2	GI|1191897829	ISLR	Immunoglobulin superfamily containing leucine rich repeat
2	GI|3024051	ITIH4	Inter-alpha-trypsin inhibitor heavy chain 4
2	GI|1191815797	ITIH5	Inter-alpha-trypsin inhibitor heavy chain 5
2	GI|83596184	KIT	KIT proto-oncogene, receptor tyrosine kinase
2	GI|1191887167	KLK6	Kallikrein related peptidase 6
2	GI|1191804379	LAMA2	Laminin subunit alpha 2
2	GI|1191911146	LAMB1	Laminin subunit beta 1
2	GI|411147409	LAMC1	Laminin subunit gamma 1
2	GI|57282794	LAMP1	Lysosomal associated membrane protein 1
2	GI|198401841	LCAT	Lecithin-cholesterol acyltransferase
2	GI|1191803369	LCN2	Lipocalin 2
2	GI|545854851	LCP1	Lymphocyte cytosolic protein 1
2	GI|1191864030	LFNG	O-fucosylpeptide 3-beta-N-acetylglucosaminyltransferase
2	GI|753703606	LGMN	Legumain
2	GI|927105033	LMAN2	Lectin, mannose binding 2
2	GI|1191801599	LOC100153854	Pancreatic alpha-amylase
2	GI|311274907	LOC100512873	Antileukoproteinase
2	GI|311267270	LOC100515166	Keratin, type I cytoskeletal 13
2	GI|1191875277	LOC100521789	Pancreatic alpha-amylase
2	GI|1191831408	LOC100623720	Collagen alpha-1
2	GI|545893416	LOC100624077	Endogenous retrovirus group V member 2 Env polyprotein
2	GI|1191848271	LOC100736623	Signal-regulatory protein beta-1-like
2	GI|937575586	LOC100738836	Insulin-like growth factor-binding protein 5
2	GI|545862104	LOC102159373	Protegrin-1
2	GI|1191848255	LOC102161654	Tyrosine-protein phosphatase non-receptor type substrate 1
2	GI|1709082	LOC110255221	Antibacterial peptide PMAP-37
2	GI|1191830205	LOC110256441	Protegrin-3
2	GI|1191799658	LOC110258046	Pancreatic alpha-amylase-like
2	GI|1191801889	LOC110258648	Protegrin-1
2	GI|1191801891	LOC110258649	Protegrin-3-like
2	GI|1191801895	LOC110258651	Protegrin-1-like
2	GI|1191899509	LOC396684	Serpin a3-5
2	GI|417515788	LRP1	LDL receptor related protein 1
2	GI|194033463	LRP11	LDL receptor related protein 11
2	GI|1191887054	LRRC4B	Leucine rich repeat containing 4B
2	GI|1191824760	LSAMP	Limbic system associated membrane protein
2	GI|303304923	LTA4H	Leukotriene A4 hydrolase
2	GI|1191885986	LTBP4	Latent transforming growth factor beta binding protein 4
2	GI|1191892917	MAG	Myelin associated glycoprotein
2	GI|1191897472	MAN2A2	Mannosidase alpha class 2A member 2
2	GI|417515707	MAN2B1	Mannosidase alpha class 2B member 1
2	GI|545848889	MCAM	Melanoma cell adhesion molecule
2	GI|1191866870	MERTK	MER proto-oncogene, tyrosine kinase
2	GI|1191823761	MFAP4	Microfibril associated protein 4
2	GI|15419710	MMP2	Matrix metallopeptidase 2
2	GI|38569737	MRC1	Mannose receptor C-type 1
2	GI|417515981	MRC2	Mannose receptor C type 2
2	GI|1191909171	NCAM1	Neural cell adhesion molecule 1
2	GI|1191830779	NCAM2	Neural cell adhesion molecule 2
2	GI|166915516	NEGR1	Neuronal growth regulator 1
2	GI|1191881533	NELL2	Neural EGFL like 2
2	GI|1191897894	NEO1	Neogenin 1
2	GI|1191910332	NFASC	Neurofascin
2	GI|1191838396	NID1	Nidogen 1
2	GI|1191809281	NID2	Nidogen 2
2	GI|1730507	NPG1	Protegrin 1
2	GI|887645	NPG3	Protegrin 3
2	GI|1006757	NPG4	Protegrin 4
2	GI|1191819667	NPTX1	Neuronal pentraxin 1
2	GI|1191864264	NPTX2	Neuronal pentraxin 2
2	GI|1191882869	NPTXR	Neuronal pentraxin receptor
2	GI|1191911181	NRCAM	Neuronal cell adhesion molecule
2	GI|1191844691	NRP2	Neuropilin 2
2	GI|1191870143	NRXN1	Neurexin 1
2	GI|1191855739	NRXN2	Neurexin 2
2	GI|1191900324	NRXN3	Neurexin 3
2	GI|1191906547	NTM	Neurotrimin
2	GI|1191875247	NTNG1	Netrin G1
2	GI|927141748	NUCB1	Nucleobindin 1
2	GI|1191909610	OAF	OUT at first homolog
2	GI|937576026	OGN	Osteoglycin
2	GI|1191812033	OLFM1	Olfactomedin 1
2	GI|311267918	OMG	Oligodendrocyte myelin glycoprotein
2	GI|1191906565	OPCML	Opioid binding protein/cell adhesion molecule like
2	GI|1191860386	PAM	Peptidylglycine alpha-amidating monooxygenase
2	GI|1191803694	PCMT1	Protein-L-isoaspartate (D-aspartate) O-methyltransferase
2	GI|335284137	PCOLCE	Procollagen C-endopeptidase enhancer
2	GI|47522896	PCSK1	Proprotein convertase subtilisin/kexin type 1
2	GI|1191914637	PCSK1N	Proprotein convertase subtilisin/kexin type 1 inhibitor
2	GI|1191833709	PEBP4	Phosphatidylethanolamine binding protein 4
2	GI|194018690	PG-2	Leukocyte antimicrobial peptide
2	GI|38605096	PGLYRP2	Peptidoglycan recognition protein 2
2	GI|1191897936	PKM	Pyruvate kinase, muscle
2	GI|1191883544	PLD3	Phospholipase D family member 3
2	GI|311258478	PLOD1	Procollagen-lysine,2-oxoglutarate 5-dioxygenase 1
2	GI|1191847641	PLTP	Phospholipid transfer protein
2	GI|1191821339	PLXDC1	Plexin domain containing 1
2	GI|1191815636	PLXDC2	Plexin domain containing 2
2	GI|1709080	PMAP-23	Antibacterial protein
2	GI|457348	PMAP-36	Antibacterial peptide
2	GI|335293565	PPIC	Peptidylprolyl isomerase C
2	GI|1191825164	PR39	Peptide antibiotic PR39
2	GI|556349	PRNP	Prion protein
2	GI|18202923	PROC	Protein C, inactivator of coagulation factors Va and VIIIa
2	GI|1191830472	PROS1	Protein S
2	GI|1191826904	PRRT3	Proline rich transmembrane protein 3
2	GI|1191833377	PSAP	Prosaposin
2	GI|343478283	PSMD2	Proteasome 26S subunit ubiquitin receptor, non-ATPase 2
2	GI|1191812895	PTPRD	Protein tyrosine phosphatase receptor type D
2	GI|1191892260	PTPRF	Protein tyrosine phosphatase receptor type F
2	GI|1191830540	PTPRG	Protein tyrosine phosphatase receptor type G
2	GI|1191841448	PTPRN	Protein tyrosine phosphatase receptor type N
2	GI|1191852712	PTPRN2	Protein tyrosine phosphatase receptor type N2
2	GI|1191858930	PTPRS	Protein tyrosine phosphatase receptor type S
2	GI|927216782	PTPRZ1	Protein tyrosine phosphatase receptor type Z1
2	GI|1191912332	QSOX1	Quiescin sulfhydryl oxidase 1
2	GI|166236191	RARRES2	Retinoic acid receptor responder 2
2	GI|526305579	RBP4	Retinol binding protein 4
2	GI|1191911075	RELN	Reelin
2	GI|311261212	RGMA	Repulsive guidance molecule BMP co-receptor a
2	GI|1191860364	RGMB	Repulsive guidance molecule BMP co-receptor b
2	GI|47116971	RNASET2	Ribonuclease T2
2	GI|350592076	ROBO1	Roundabout guidance receptor 1
2	GI|195969549	S100A12	s100 calcium binding protein A12
2	GI|1191875159	SARS1	Seryl-tRNA synthetase 1
2	GI|1191893693	SDF4	Stromal cell derived factor 4
2	GI|194036227	SELENBP1	Selenium binding protein 1
2	GI|1191870611	SELL	Selectin L
2	GI|194041227	SEMA3G	Semaphorin 3G
2	GI|1191897587	SEMA4B	Semaphorin 4B
2	GI|1191897797	SEMA7A	Semaphorin 7A
2	GI|1703026	SERPINA1	Serpin family A member 1
2	GI|9955853	SERPINA3-2	Alpha-1-antichymotrypsin 2
2	GI|48237475	SERPINA7	Serpin family A member 7
2	GI|194043402	SERPIND1	Serpin family D member 1
2	GI|178056710	SERPING1	Serpin family G member 1
2	GI|1191828056	SERPINI1	Serpin family I member 1
2	GI|1191822569	SEZ6	Seizure related 6 homolog
2	GI|1191865347	SEZ6L2	Seizure related 6 homolog like 2
2	GI|1191905668	SGCE	Sarcoglycan epsilon
2	GI|1191847716	SIRPA	Signal regulatory protein alpha
2	GI|115522015	SOD3	Superoxide dismutase 3
2	GI|1191835824	SORCS1	Sortilin related VPS10 domain containing receptor 1
2	GI|1191902897	SORCS2	Sortilin related VPS10 domain containing receptor 2
2	GI|927124531	SORT1	Sortilin 1
2	GI|138752624	SPARC	Secreted protein acidic and cysteine rich
2	GI|545846755	SPARCL1	SPARC like 1
2	GI|1191836837	SPOCK2	SPARC (osteonectin), cwcv and kazal like domains proteoglycan 2
2	GI|1191857018	SPON1	Spondin 1
2	GI|129262	SPP1	Secreted phosphoprotein 1
2	GI|1191849008	SULF2	Sulfatase 2
2	GI|1191811894	SURF6	Surfeit 6
2	GI|349732238	TALDO1	Transaldolase 1
2	GI|1191813959	TGFB2	Transforming growth factor beta 2
2	GI|1191861030	TGFBI	Transforming growth factor beta induced
2	GI|1191871284	TGFBR3	Transforming growth factor beta receptor 3
2	GI|1191860164	THBS4	Thrombospondin 4
2	GI|6572666	TIMP2	Timp metallopeptidase inhibitor 2
2	GI|1191856082	TMEM132A	Transmembrane protein 132A
2	GI|87047642	TMSB4X	Thymosin beta 4 X-linked
2	GI|1191804116	TNC	Tenascin C
2	GI|335294265	TPP1	Tripeptidyl peptidase 1
2	GI|1016032	TTR	Transthyretin
2	GI|47522704	VCAM1	Vascular cell adhesion molecule 1
2	GI|1351418	VTN	Vitronectin
2	GI|1191877936	VWF	Von Willebrand factor
2	GI|1191821714	WFIKKN2	WAP, follistatin/kazal, immunoglobulin, kunitz and netrin domain containing 2
3	GI|257467621	ACAN	Aggrecan
3	GI|335289612	ACTN4	Actinin alpha 4
3	GI|1191855923	AHNAK	AHNAK nucleoprotein
3	GI|1184820	AKR1B1	Aldo-keto reductase family 1 member B
3	GI|1191873380	ALDH9A1	Aldehyde dehydrogenase 9 family member A1
3	GI|54020966	ANXA2	Annexin A2
3	GI|335293906	ANXA5	Annexin A5
3	GI|1191886929	AP2A1	Adaptor related protein complex 2 subunit alpha 1
3	GI|1191818741	ARHGDIA	Rho GDP dissociation inhibitor alpha
3	GI|1191812960	ASTN2	Astrotactin 2
3	GI|335289705	BLVRB	Biliverdin reductase B
3	GI|927179111	C4BPB	Complement component 4 binding protein beta
3	GI|194037099	CA1	Carbonic anhydrase 1
3	GI|1191852730	CALD1	Caldesmon 1
3	GI|1191821428	CDK5RAP3	CDK5 regulatory subunit associated protein 3
3	GI|545858654	CHAD	Chondroadherin
3	GI|348605274	ADH5	Alcohol dehydrogenase 5
3	GI|285818436	COMT	Catechol-O-methyltransferase
3	GI|148470135	CRABP1	Cellular retinoic acid binding protein 1
3	GI|335296245	CSRP1	Cysteine and glycine rich protein 1
3	GI|6166051	CYB5A	Cytochrome b5 type A
3	GI|1191875896	DDAH1	Dimethylarginine dimethylaminohydrolase 1
3	GI|1191837913	DPYSL2	Dihydropyrimidinase like 2
3	GI|417515810	EEA1	Early endosome antigen 1
3	GI|110287842	EEF1A1	Eukaryotic translation elongation factor 1 alpha 1
3	GI|1191855932	EEF1G	Eukaryotic translation elongation factor 1 gamma
3	GI|335282386	EEF2	Eukaryotic translation elongation factor 2
3	GI|1191823396	EIF5A	Eukaryotic translation initiation factor 5A
3	GI|117938482	FABP4	Fatty acid binding protein 4
3	GI|927141869	FCGRT	Fc gamma receptor and transporter
3	GI|120094	FGA	Fibrinogen alpha chain
3	GI|1191916144	FLNA	Filamin A
3	GI|11611545	GALM	Galactose mutarotase
3	GI|75043802	GDI2	GDP dissociation inhibitor 2
3	GI|927106502	GNPDA1	Glucosamine-6-phosphate deaminase 1
3	GI|12249197	GSTO1	Glutathione S-transferase omega 1
3	GI|66775665	H1-2	H1.2 linker histone, cluster member
3	GI|1191831165	HMGN1	High mobility group nucleosome binding domain 1
3	GI|545825488	HNRNPA1	Heterogeneous nuclear ribonucleoprotein A1
3	GI|545883566	HNRNPA2B1	Heterogeneous nuclear ribonucleoprotein A2/B1
3	GI|1191841860	HNRNPA3	Heterogeneous nuclear ribonucleoprotein A3
3	GI|1191905446	HNRNPD	Heterogeneous nuclear ribonucleoprotein D
3	GI|1191813344	HNRNPK	Heterogeneous nuclear ribonucleoprotein K
3	GI|39777368	HSP70.2	Heat shock protein 70.2
3	GI|55926209	HSPB1	Heat shock protein family B
3	GI|41323579	IGKV	Immunoglobulin kappa variable group
3	GI|1191901134	KIF13A	Kinesin family member 13A
3	GI|1191821122	KRT15	Keratin 15
3	GI|350590334	KRT25	Keratin 25
3	GI|1191903162	LAP3	Leucine aminopeptidase 3
3	GI|1191821346	LASP1	LIM and SH3 protein 1
3	GI|343790977	LHPP	Phospholysine phosphohistidine inorganic pyrophosphate phosphatase
3	GI|1191870603	LMNA	Lamin A/C
3	GI|1191907911	LOC100515788	Hemoglobin subunit beta-like
3	GI|45479846	LOC595122	Histone H1.3-like protein
3	GI|1191830135	MAP4	Microtubule associated protein 4
3	GI|124075357	MIF	Macrophage migration inhibitory factor
3	GI|127236	MSN	Moesin
3	GI|1191890277	MTCL1	Microtubule crosslinking factor 1
3	GI|1191829258	MYLK	Myosin light chain kinase
3	GI|115311824	NME2	NME/NM23 nucleoside diphosphate kinase 2
3	GI|358009193	P4HB	Prolyl 4-hydroxylase subunit beta
3	GI|1191861470	PCDH1	Protocadherin 1
3	GI|545866417	PCNP	PEST proteolytic signal containing nuclear protein
3	GI|1191864516	PDXDC1	Pyridoxal dependent decarboxylase domain containing 1
3	GI|545864363	PFN2	Profilin 2
3	GI|753703322	PLCL2	Phospholipase C like 2
3	GI|1191916461	PLS3	Plastin 3
3	GI|510469	PPP2R1A	Protein phosphatase 2 scaffold subunit Aalpha
3	GI|1191891998	PRDX1	Peroxiredoxin 1
3	GI|545884303	PRDX4	Peroxiredoxin 4
3	GI|417515732	PSMA2	Proteasome 20S subunit alpha 2
3	GI|194034201	PSMA3	Proteasome 20S subunit alpha 3
3	GI|1191871359	PSMA5	Proteasome 20S subunit alpha 5
3	GI|1191881508	PUS7L	Pseudouridine synthase 7 like
3	GI|115394758	RAB1B	RAB1B, member RAS oncogene family
3	GI|495144	RACK1	Receptor for activated C kinase 1
3	GI|1191835373	RAN	RAN, Member RAS oncogene family
3	GI|1191873107	RB1CC1	RB1 inducible coiled-coil 1
3	GI|545840664	RCN2	Reticulocalbin 2
3	GI|1191849615	RRBP1	Ribosome binding protein 1
3	GI|1191835321	RYR2	Ryanodine receptor 2
3	GI|345091075	SEC61B	SEC61 translocon subunit beta
3	GI|55742799	SERPINA6	Serpin family A member 6
3	GI|346421378	SERPINH1	Serpin family H member 1
3	GI|1191820793	SLC4A1	Solute carrier family 4 member 1
3	GI|66269674	SLC4A4	Solute carrier family 4 member 4
3	GI|545848661	TAGLN	Transgelin
3	GI|335286672	TAGLN2	Transgelin 2
3	GI|1191911447	TNN	Tenascin N
3	GI|1191803207	TPM1	Tropomyosin 1
3	GI|262263207	TPM4	Tropomyosin 4
3	GI|47523802	TPT1	Tumor protein, translationally-controlled 1
3	GI|1191843891	TTN	Titin
3	GI|326633214	UBE2V1	Ubiquitin conjugating enzyme E2 V1
3	GI|38492203	UCHL1	Ubiquitin C-terminal hydrolase L1
3	GI|1174636	VCP	Valosin containing protein
3	GI|1191903022	WDR1	WD repeat domain 1
3	GI|354549569	YBX1	Y-box binding protein 1
4	GI|1191880959	A2M	Alpha-2-macroglobulin
4	GI|1191864135	ACTB	Actin beta
4	GI|335297229	ACTG1	Actin gamma 1
4	GI|340007404	ACTN1	Actinin alpha 1
4	GI|73811113	ADIPOQ	Adiponectin, C1Q and collagen domain containing
4	GI|1191823302	ARRB2	Arrestin beta 2
4	GI|1191840382	ATG9A	Autophagy related 9A
4	GI|194037097	CA2	Carbonic anhydrase 2
4	GI|346644746	CALM1	Calmodulin 1
4	GI|311252670	CALM2	Calmodulin 2
4	GI|2654179	CALM3	Calmodulin 3
4	GI|350595262	CALU	Calumenin
4	GI|1191890087	CAP1	Cyclase associated actin cytoskeleton regulatory protein 1
4	GI|356460899	CAT	Catalase
4	GI|1191801660	CFH	COMPLEMENT factor H
4	GI|51592135	CFL1	Cofilin 1
4	GI|1279695551	CKB	Creatine kinase B
4	GI|1191888349	CLSTN1	Calsyntenin 1
4	GI|1191806488	COL12A1	Collagen type XII alpha 1 chain
4	GI|62086218	COL5A2	Collagen type V alpha 2 chain
4	GI|1191828842	CPN2	Carboxypeptidase N subunit 2
4	GI|134290405	CSTB	Cystatin B
4	GI|1191888305	ENO1	Enolase 1
4	GI|1191801669	F13B	Coagulation factor XIII B chain
4	GI|60203061	FABP5	Fatty acid binding protein 5
4	GI|47523126	FCN2	Ficolin
4	GI|1191901997	FRAS1	Fraser extracellular matrix complex subunit 1
4	GI|226372953	FSCN1	Fascin actin-bundling protein 1
4	GI|122142902	GNA11	G protein subunit alpha 11
4	GI|1364248	GPI	Glucose-6-phosphate isomerase
4	GI|1191900009	H1-5	H1.5 linker histone, cluster member
4	GI|261245058	HBB	Hemoglobin, beta
4	GI|1191827257	DNAJC13	DnaJ heat shock protein family Hsp40 member C13
4	GI|462328	HSPA6	Heat shock protein family A6
4	GI|345441750	HSPA8	Heat shock protein family A8
4	GI|335293621	JCHAIN	Joining chain of multimeric IgA and IgM
4	GI|1191875854	KYAT3	Kynurenine aminotransferase 3
4	GI|1170740	LDHA	LACTATE dehydrogenase A
4	GI|62530180	LGALS1	Galectin 1
4	GI|927205973	LOC100155138	Tubulin alpha-3 chain
4	GI|1191843505	LOC100158003	Tubulin alpha-1D chain
4	GI|311271065	LOC100510930	Tubulin alpha-3 chain
4	GI|1191866723	LOC100737768	Hemoglobin subunit alpha-like
4	GI|1191800842	LOC110258323	Protein S100-B-like
4	GI|1191866721	LOC110259958	Hemoglobin subunit alpha
4	GI|1191875079	LOC110260348	Glutathione S-transferase Mu 1-like
4	GI|343183420	LUM	Lumican
4	GI|1191841519	MAP2	Microtubule associated protein 2
4	GI|1191806273	MARCKS	Myristoylated alanine rich protein kinase C substrate
4	GI|2497785	ME1	Malic enzyme 1
4	GI|1191852566	MGAM	Maltase-glucoamylase
4	GI|24397195	MYOC	Myocilin
4	GI|1191909177	NCAM1	Neural cell adhesion molecule 1
4	GI|113374885	NME1	NME/NM23 nucleoside diphosphate kinase 1
4	GI|67038668	PARK7	Parkinsonism associated deglycase
4	GI|301016769	PDIA3	Protein disulfide isomerase family A member 3
4	GI|927138517	PEPD	Peptidase D
4	GI|262204914	PGK1	Phosphoglycerate kinase 1
4	GI|1191883917	PGM1	Phosphoglucomutase 1
4	GI|545854580	POSTN	Periostin
4	GI|927217413	PPIA	Peptidylprolyl isomerase A
4	GI|1191806991	PPIB	Peptidylprolyl isomerase B
4	GI|1717797	PRDX2	Peroxiredoxin 2
4	GI|75074817	PRDX6	Peroxiredoxin 6
4	GI|346986249	PSMB1	Proteasome 20S subunit beta 1
4	GI|1191880955	PZP	Pregnancy zone protein
4	GI|927141966	RCN3	Reticulocalbin 3
4	GI|1191874299	S100A1	S100 calcium binding protein A1
4	GI|1191803128	S100B	S100 calcium binding protein B
4	GI|460417876	SFN	Stratifin
4	GI|1191889177	SH3BGRL3	SH3 domain binding glutamate rich protein like 3
4	GI|159502444	TKT	Transketolase
4	GI|148231384	TMSB10	Thymosin beta 10
4	GI|80971510	TPI1	Triosephosphate isomerase 1
4	GI|1191871309	TPM3	Tropomyosin 3
4	GI|545824995	TUBA1A	Tubulin alpha 1a
4	GI|1191900919	TXNDC5	Thioredoxin domain containing 5
4	GI|47522618	VCL	Vinculin
4	GI|545853401	VIM	Vimentin
4	GI|545880805	YWHAB	Tyrosine 3-monooxygenase/tryptophan 5-monooxygenase activation protein beta
4	GI|1191822700	YWHAE	Tyrosine 3-monooxygenase/tryptophan 5-monooxygenase activation protein epsilon
4	GI|545813611	YWHAG	Tyrosine 3-monooxygenase/tryptophan 5-monooxygenase activation protein gamma
4	GI|194043292	YWHAH	Tyrosine 3-monooxygenase/tryptophan 5-monooxygenase activation protein eta
4	GI|138753471	YWHAQ	Tyrosine 3-monooxygenase/tryptophan 5-monooxygenase activation protein theta
4	GI|350583022	YWHAZ	Tyrosine 3-monooxygenase/tryptophan 5-monooxygenase activation protein zeta

Several proteins and biological responses stand out as stimulation-induced factors. The inflammatory cytokine IL6 was below detection limits in whole tissue ISF, CSF, and the first two perfusate epochs but was detected at 1.8 × 10^6^ units in epoch 3 and 5.5 × 10^6^ units in epoch 4. Likewise, the inflammatory protease MMP9 was below detection in ISF, CSF, and the first two epochs but was then detected at 2.5 × 10^7^ units in epoch 3 and 6.5 × 10^7^ units in epoch 4. The inflammatory stress proteins S100A8 and S100A9, which together form the calprotectin complex, were both below detection in ISF, CSF, and the first two epochs but were then detected at 1.4 × 10^7^ and 2.9 × 10^6^ units in epoch 3 and 4.9 × 10^7^ and 3.8 × 10^6^ units in epoch 4, respectively. Another notable response was observed in the family of complement factor proteins (figure 5(E)). In the post-stimulation epoch (epoch 3) there was a clear increase in levels of nearly every component of the complement cascade, suggesting that stimulation drove the active release and processing of these factors.

## 4. Discussion

We describe the development of a modified brain microperfusion-EEG electrode device that uses off-the-shelf subassemblies. The Ad-Tech Spencer depth electrode, the M Dialysis AB fluidics assembly, and the M Dialysis AB adjustable flow rate pump are each approved for human use and are provided in sterile surgery-ready packaging. We are currently exploring the biocompatibility and sterility validation necessary for human use of the integrated system presented herein. Our findings support the use of this device for performing powerful unbiased proteomic analyses of perfusate collected from the brain during seizures and our near-term goal will be to gather such data from patients undergoing resection surgery for drug-resistant epilepsy.

The use of existing components allowed us to bypass the lengthy and costly process of developing new devices. We realized that the open lumen present in the Ad-Tech Spencer electrode was appropriately sized to accept the M Dialysis 71 microdialysis membrane and fluidics assembly. We further realized that precision ablation of fluid entry ports into the Spencer depth electrode would permit direct exchange of materials from the ISF surrounding the electrode into the lumen and across the dialysis membrane. In our initial tests we focused on cleanly ablating holes into the electrode body around the second-most distal macroelectrode. We identified the optimal laser power, focusing, and beam pitch parameters yielding clean, reproducible pores and we validated the maintenance of electrical connections through the electrode following ablation. While impedance was altered following ablation, we note that the decay was identical across all four macroelectrodes, an outcome that suggests a systemic change to the function of the electrode rather than a specific process of damage to the wires carrying current. Indeed, the wires emanating from the two macroelectrodes located above the modified macroelectrode do not pass through the polymer sheath at the site of ablation and therefore could not have been damaged by the laser. Importantly, despite the impedance changes associated with fluid access to the lumen of the electrode, electrochemical impedance spectroscopy across a physiologically relevant frequency band revealed no appreciable differences in the modified electrode response profile. Moreover, the modified probe did not exhibit degradation in signal-to-noise performance.

During the course of developing the device we recognized the potential value in removing the microdialysis membrane. As we have discussed elsewhere [1], microperfusion offers significant advantages with regard to an unbiased sampling of the ISF. In addition to removing the confounding and unpredictably stochastic nature of the molecular weight cutoff inherent to microdialysis membranes, running the device in open microperfusion mode offers the ability to capture large molecular weight macromolecules and large membrane-bound structures such as extracellular vesicles. Therefore, in addition to modifying the electrode to permit fluid flow, we also modified the M Dialysis 107 pump to operate in a push–pull manner. In order to prevent net fluid loss from the perfusate into the surrounding tissue, the flow rate through the perfusion space must be maintained and controlled by both a push pump and a pull pump acting simultaneously. The dual pump modification resulted in reproducible recovery of test analytes from fluid and agar substrates, including recovery of more than 70% of large molecular weight dextran from agar during a short collection window. In comparison, recovery of a 66 kDa protein by microdialysis using a 100 kDa membrane was less than 1% in the first hour of collection and recovery rapidly decreased thereafter due to membrane fouling [31].

The unbiased nature of collection in our system is highlighted by the recovery of numerous very large molecular weight proteins and by the unique detection of multiple inflammatory proteins following electrical stimulation at the macroelectrode coincident with microperfusion pores. We used intensity-based absolute quantification of proteins [26] to measure tryptic peptide concentrations in perfusates from each collection epoch and in CSF and ISF collected from the same animal. Albumin is a known protein constituent of CSF that is present at approximately 0.1 mg ml^−1^ under healthy conditions [32]. We measured 2 × 10^10^ iBAQ units of albumin in CSF and a remarkably consistent level of albumin in perfusate from all four epochs (1.5 × 10^10^, 1.7 × 10^10^, 1.6 × 10^10^, 1.2 × 10^10^ respectively), providing confidence that the perfusates represented ISF. Using this metric as a quantitative unit to calculate estimated molar concentrations of other proteins measured in the samples, we found that IL6, which was undetected in CSF and in the initial baseline epoch, was detected at approximately 0.5 nM in perfusate from the post-stimulation epoch. This concentration is consistent with the amount of IL6 released by stimulated microglia [33] and suggests that electrical pulsing at 2 Hz during the previous epoch induced released of IL6 that was detectable and quantifiable using our microperfusion electrode device. Moreover, given that IL6 was not detected in bulk ISF collected from the tissue surrounding the probe implantation site, the IL6 detected in epoch 3 was likely restricted to a constrained spatial domain around the stimulated electrode. Our device may therefore provide unprecedented insight into the proteomic response induced within tissue localized to a seizure focus.

## 5. Conclusions

**Figure 6.**
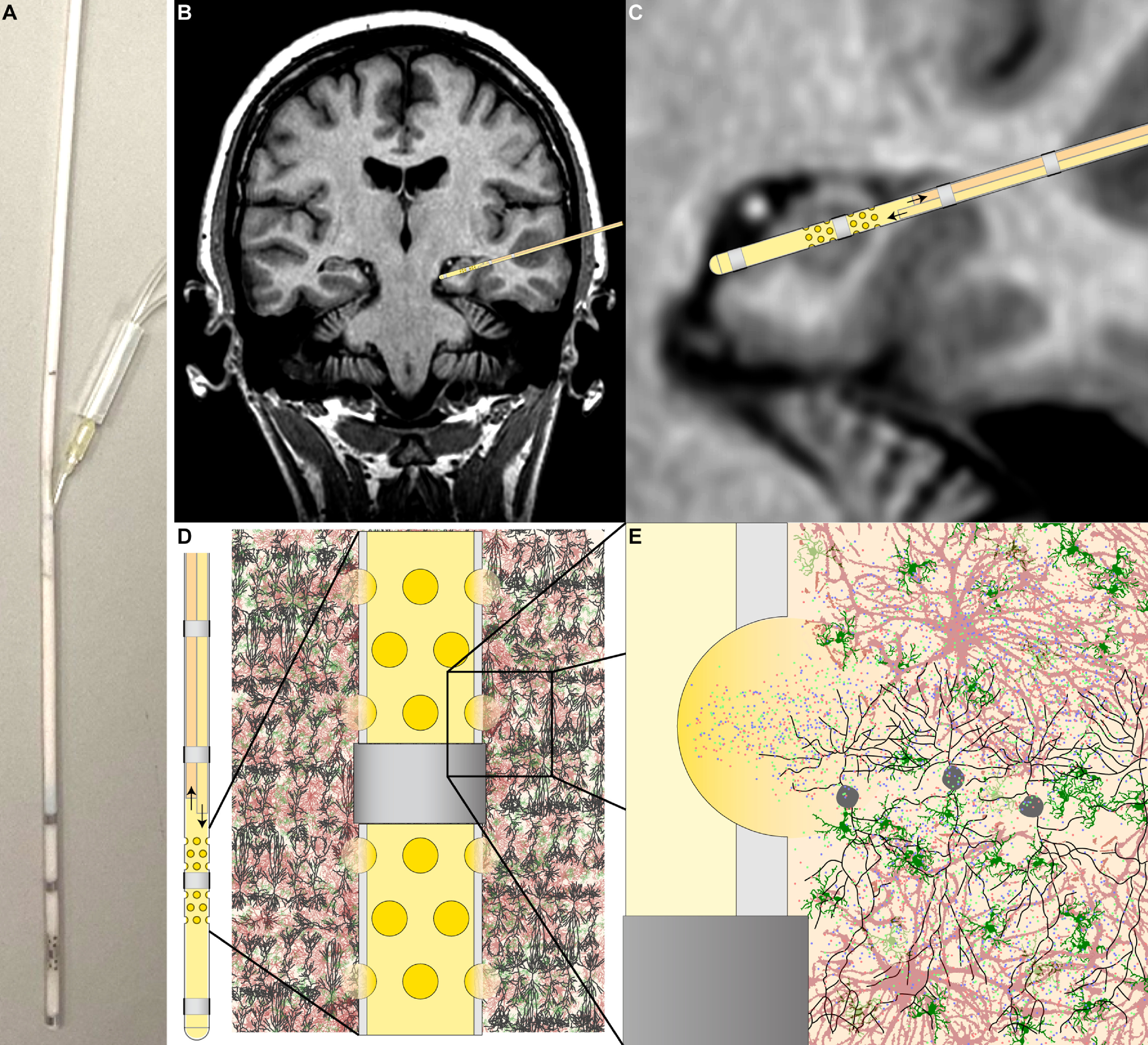
Schematic overview of the microperfusion-EEG dual-sensing probe. (A) Representative image of a modified depth electrode with associated fluidics. (B) Hypothetical trajectory for electrode implantation into the human hippocampus. (C) Hypothetical localization of the microperfusion ports and macroelectrode in the human hippocampus. (D) Schematic of the modified dual-sensing electrode showing the scale of the microperfusion ports to the surrounding tissue. Pyramidal neurons in black, astrocytes in red, microglia in green. (E) Expanded schematic view showing the overall concept and proposed microperfusion sampling of the extracellular fluid compartment around neurons and glia proximal to the macroelectrode.

Laser ablation of an off-the-shelf Spencer clinical depth electrode coupled with push–pull pump modification of an off-the-shelf perfusion pump system and fluidics resulted in a device that permits coincident detection of electrophysiological changes and unbiased proteomic changes within a highly localized spatial domain in the brain of a living organism. This device recovered high molecular weight proteins with excellent efficiency and revealed stimulation-induced changes in the extracellular proteome that were not detectable in bulk tissue ISF from the stimulation site. Ongoing experiments in large non-human animal models of epilepsy include tuning the flow rate to increase detection of low-abundance proteins (by reducing total volume of the perfusate) or to increase temporal resolution (by shortening the collection epoch), employing additional enrichment steps upstream from the proteomics analysis, and collecting perfusates from non-stimulated control electrodes to discriminate stimulation-induced responses from innate immune responses to the implanted device. Based on the promising findings described herein, our future goal will be to stereotactically implant the microperfusion electrode (figure 6) into the ictogenic zone of patients with drug-resistant epilepsy undergoing assessment for resection surgery and correlate localized EEG changes measured on the macroelectrode at the site of the microperfusion pores with proteomic changes measured by unbiased mass spectrometry of the perfusate. Our current findings strongly support the use of coincident EEG and microperfusion as a powerful tool for uncovering pathogenic mechanisms operating at spatial scales that are inaccessible to current bulk tissue methods.

## Data Availability

The data that support the findings of this study are available upon reasonable request from the authors.
